# Assembly-dependent translation of subunits *6* (Atp6) and *9* (Atp9) of ATP synthase in yeast mitochondria

**DOI:** 10.1093/genetics/iyac007

**Published:** 2022-01-20

**Authors:** Anna M Kabala, Krystyna Binko, François Godard, Camille Charles, Alain Dautant, Emilia Baranowska, Natalia Skoczen, Kewin Gombeau, Marine Bouhier, Hubert D Becker, Sharon H Ackerman, Lars M Steinmetz, Déborah Tribouillard-Tanvier, Roza Kucharczyk, Jean-Paul di Rago

**Affiliations:** CNRS, IBGC, University of Bordeaux, UMR 5095, F-33000 Bordeaux, France; Institute of Biochemistry and Biophysics, Polish Academy of Sciences, 01-224 Warsaw, Poland; CNRS, IBGC, University of Bordeaux, UMR 5095, F-33000 Bordeaux, France; Institute of Biochemistry and Biophysics, Polish Academy of Sciences, 01-224 Warsaw, Poland; CNRS, IBGC, University of Bordeaux, UMR 5095, F-33000 Bordeaux, France; CNRS, IBGC, University of Bordeaux, UMR 5095, F-33000 Bordeaux, France; CNRS, IBGC, University of Bordeaux, UMR 5095, F-33000 Bordeaux, France; Institute of Biochemistry and Biophysics, Polish Academy of Sciences, 01-224 Warsaw, Poland; CNRS, IBGC, University of Bordeaux, UMR 5095, F-33000 Bordeaux, France; Institute of Biochemistry and Biophysics, Polish Academy of Sciences, 01-224 Warsaw, Poland; CNRS, IBGC, University of Bordeaux, UMR 5095, F-33000 Bordeaux, France; CNRS, IBGC, University of Bordeaux, UMR 5095, F-33000 Bordeaux, France; UPR ‘Architecture et Réactivité de l’ARN’, CNRS, Institut de Biologie Moléculaire et Cellulaire, Université de Strasbourg, F-67084 Strasbourg Cedex, France; Department of Biochemistry and Molecular Biology, Wayne State University School of Medicine, Detroit, MI 48202, USA; European Molecular Biology Laboratory (EMBL), Genome Biology Unit, 69117 Heidelberg, Germany; Department of Genetics, Stanford University School of Medicine, Stanford, CA 94305, USA; Stanford Genome Technology Center, Palo Alto, CA 94304, USA; CNRS, IBGC, University of Bordeaux, UMR 5095, F-33000 Bordeaux, France; Institute of Biochemistry and Biophysics, Polish Academy of Sciences, 01-224 Warsaw, Poland; CNRS, IBGC, University of Bordeaux, UMR 5095, F-33000 Bordeaux, France

**Keywords:** ATP synthase, mitochondria, mitochondrial biogenesis, mitochondria DNA, yeast, mitochondrial gene expression

## Abstract

The yeast mitochondrial ATP synthase is an assembly of 28 subunits of 17 types of which 3 (subunits *6*, *8*, and *9*) are encoded by mitochondrial genes, while the 14 others have a nuclear genetic origin. Within the membrane domain (F_O_) of this enzyme, the subunit *6* and a ring of 10 identical subunits *9* transport protons across the mitochondrial inner membrane coupled to ATP synthesis in the extra-membrane structure (F_1_) of ATP synthase. As a result of their dual genetic origin, the ATP synthase subunits are synthesized in the cytosol and inside the mitochondrion. How they are produced in the proper stoichiometry from two different cellular compartments is still poorly understood. The experiments herein reported show that the rate of translation of the subunits *9* and *6* is enhanced in strains with mutations leading to specific defects in the assembly of these proteins. These translation modifications involve assembly intermediates interacting with subunits *6* and *9* within the final enzyme and *cis*-regulatory sequences that control gene expression in the organelle. In addition to enabling a balanced output of the ATP synthase subunits, these assembly-dependent feedback loops are presumably important to limit the accumulation of harmful assembly intermediates that have the potential to dissipate the mitochondrial membrane electrical potential and the main source of chemical energy of the cell.

## Introduction

Mitochondria support aerobic respiration and produce the bulk of cellular ATP through the process of oxidative phosphorylation (OXPHOS) (Saraste 1999). Typically, OXPHOS involves 5 hetero-oligomeric protein complexes (numbered I–V) that are located in the mitochondrial inner membrane. Complexes I–IV transfer to oxygen electrons from carbohydrates and fatty acids coupled to the transport of protons from the mitochondrial matrix to the intermembrane space, and the resulting energy-rich transmembrane proton gradient is used by the Complex V (ATP synthase) to make ATP from ADP and inorganic phosphate.

The structural genes of the mitochondrial OXPHOS complexes are located in nuclear and mitochondrial DNA. As such, 2 separate translation machineries, one cytosolic and the other inside the mitochondrion, are utilized to synthesize their gene products (Ott and Herrmann 2010). A plethora of proteins that do not belong to the OXPHOS complexes assist translation and assembly of their subunits in *Saccharomyces cerevisiae* (Fox 1996, 2012; Herrmann *et al.* 2013; Ott *et al.* 2016). A subset of these “helper” proteins are known to interact with the 5′-UTR (Untranslated Region) of a specific mitochondrial mRNA transcript, and in some instances, with the translation product as well, and these observations have led investigators to postulate the existence of regulatory feedback loops that couple translation and assembly of mitochondrial gene products (Fox 2012; Fontanesi 2013; Herrmann *et al.* 2013; Ott *et al.* 2016). The most thoroughly investigated example is Mss51, which facilitates Cox1 translation and assembly in complex IV (Soto *et al.* 2012). Studies have shown that Mss51 binds first to the 5′-UTR of the *COX1* mRNA, where it activates translation, and then to the newly synthesized Cox1 protein until Cox1 is assembled with its partner subunits (Perez-Martinez *et al.* 2003; Barrientos *et al.* 2004; Pierrel *et al.* 2007; Mick *et al.* 2010). The posttranslational activity of Mss51 is similar to that described for another mitochondrial protein, which is a small complex composed of Cbp3 and Cbp6 polypeptides that maintains association with newly translated cytochrome *b* through its acquisition of heme cofactors and assembly with the nucleus-encoded subunits of complex III (Rodel 1986; Chen and Dieckmann 1994; Dieckmann and Staples 1994; Gruschke *et al.* 2011; Hildenbeutel *et al.* 2014). Similarly, the Sov1 protein assists in yeast the translational activation and assembly of the mtDNA-encoded Var1 subunit of the mitochondrial ribosome (Seshadri *et al.* 2020).

Much less is known about the regulation of yeast ATP synthase biogenesis. This is an assembly of 28 subunits of 17 types that are encoded by 3 mitochondrial (*ATP6*, *ATP8*, and *ATP9*) and 14 nuclear genes. This enzyme, known as the F_1_F_O_ complex, is organized into a largely hydrophobic domain (F_O_) that transports protons through the membrane, and a hydrophilic domain (F_1_) in the mitochondrial matrix where ATP is synthesized. The *ATP6* and *ATP9* genes encode subunits of the F_O_ (*6*/*a* and *9*/*c*) that form an integral proton channel made of one subunit *6* and an oligomeric ring of 10 subunits *9* (*9*_10_-ring). During proton translocation, the *9*_10_-ring rotates, and this induces conformational changes in the F_1_ that promote ATP synthesis. The *ATP8* gene encodes a membrane-embedded protein (the subunit *8*) that stabilizes the proton channel of ATP synthase. *ATP9* is co-transcribed with tRNA^ser^ and a mitochondrial ribosome subunit gene (*VAR1*), whereas *ATP8* and *ATP6* are co-transcribed with *COX1* (Christianson and Rabinowitz 1983; Zassenhaus *et al.* 1984; Finnegan *et al.* 1991). Processing of the primary transcripts produces a major *ATP9* mRNA with a 0.63 kb long 5′-UTR, and near equal amounts of 2 bicistronic *ATP8*,*6* mRNAs that differ by the length of their 5′-UTR (Foury *et al.* 1998).

Among the proteins that are associated with the biogenesis pathway for subunits *6*, *8*, and *9*, four (Nca2, Nca3, Aep3, and Nam1) contribute to *ATP8*/*6* transcripts stability (Asher *et al.* 1989; Pelissier *et al.* 1992, 1995; Ellis *et al.* 2004). A role for Aep3 in translation of subunit *8* has also been reported (Barros and Tzagoloff 2017). Other proteins include Atp22, which activates subunit *6* translation (Zeng *et al.* 2007a), and Smt1, which represses translation of both subunits *6* and *8* (Rak *et al.* 2016). The stability of the *ATP9* transcript is dependent on a 35 kDa C-terminal cleavage fragment of Atp25 (Zeng *et al.* 2008) that is released in the matrix by the mitochondrial processing peptidase (Woellhaf *et al.* 2016). Two more proteins with activities linked to the *ATP9* mRNA are Aep1 and Aep2 (Ackerman *et al.* 1991; Finnegan *et al.* 1991, 1995; Payne *et al.* 1991, 1993; Ziaja *et al.* 1993). Some studies have suggested a role for these proteins in *ATP9* mRNA stability/processing (Payne *et al.* 1991; Ziaja *et al.* 1993), while others have led investigators to propose that they activate translation (Payne *et al.* 1993; Ellis *et al.* 2004).

Another subgroup of yeast nuclear gene products associated with ATP synthase biogenesis assists oligomerization of its subunits. Two such proteins, Atp11 and Atp12, are essential for creating the [αβ]_3_ hexamer, which houses the catalytic sites for ATP synthesis and contributes ∼85% of the mass in F_1_ (Ackerman and Tzagoloff 1990b; Wang and Ackerman 2000; Wang *et al.* 2000, 2001; Ackerman 2002; Lefebvre-Legendre *et al.* 2005). A third protein that is part of this process, Fmc1, is necessary for Atp12 folding/stability at elevated temperature (36°C) (Lefebvre-Legendre *et al.* 2001, 2005). Other work has shown that a complex of 2 proteins, Ina22 and Ina17, facilitates assembly of ATP synthase peripheral stalk, which is a structure protruding from the membrane and that contacts the surface of the F_1_ to prevent rotation of the [αβ]_3_ hexamer during catalysis (Lytovchenko *et al.* 2014). In an early model of F_1_F_O_ assembly, the *9*_10_-ring was proposed to assemble spontaneously (Arechaga *et al.* 2002). This was challenged by later studies suggesting that oligomerization of the *9*_10_-ring is chaperoned by a 32 kDa N-terminal fragment of Atp25 (Zeng *et al.* 2008). This fragment is homologous to the bacterial ribosome-silencing factor and was found associated with the mitochondrial ribosome (Woellhaf *et al.* 2016). Two proteins (Atp10 and Atp23) have been assigned roles in procuring the assembly/stability of subunit *6* (Paul *et al.* 2000; Tzagoloff *et al.* 2004; Osman *et al.* 2007; Zeng *et al.* 2007c). Atp10 was shown to associate in a physical complex with newly translated subunit *6* and promote a favorable interaction with the *9*_10_-ring (Tzagoloff *et al.* 2004). Atp23 was identified to be a protease that cleaves the first 10 amino acid residues of the nascent subunit *6* polypeptide and has been linked also to folding the processed protein (Osman *et al.* 2007; Zeng *et al.* 2007b, 2007c). Finally, the interaction of subunit *6* with the *9*_10_-ring has been reported to depend on Oxa1 (Jia *et al.* 2007), which is a protein translocase involved in the insertion of certain mitochondrial and nuclear gene products into the inner membrane (Altamura *et al.* 1996; Herrmann *et al.* 1997; Hell *et al.* 2001).

Herein we report that translation of subunit *6* and *9* is enhanced in mutant strains with specific defects in the assembly of these proteins. These translation modifications involve assembly intermediates interacting with these proteins within the final ATP synthase and *cis*-regulatory sequences that control gene expression in the organelle. These findings suggest that the subunit *6* is part of an assembly-dependent feedback loop that is different from a previously reported regulatory model for this protein (Rak *et al.* 2009, 2011). Additionally, they contradict the generally accepted view that the *9*_10_-ring forms separately, independently of any other ATP synthase component.

## Materials and methods

### Growth media

The media used for growing yeast were: YPGA (rich glucose): 1% Bacto yeast extract, 1% Bacto peptone, 2% glucose, 40 mg/L adenine; YPGalA (rich galactose): 1% Bacto yeast extract, 1% Bacto Peptone, 2% galactose, 40 mg/L adenine; YPGlyA (rich glycerol): 1% Bacto yeast extract, 1% Bacto peptone, 2% glycerol, 3% ethanol, 40 mg/L adenine; complete synthetic medium (CSM) lacking 1 amino acid (arginine, leucine, or tryptophan) or base (uracil): glucose or galactose 20 g/L, yeast nitrogen base 1.7 g/L, ammonium sulfate 5 g/L, adenine 40 mg/L, and CSM single drop-out powder 0.74-0.77 g/L (see MB Biochemicals for instruction). The liquid media were solidified with 2% of Bacto agar. Doxycycline was used at the concentration of 40 µg/mL. G418 sulfate was used at 200 µg/mL.

### Construction and isolation of yeast strains

The genotypes of strains used in this study are listed in Table 1. All the mutant strains are derivatives of wild-type strain MR6 (Rak *et al.* 2007b). Deletion of *AEP1*, *AEP2*, and *ATP25* in wild-type strain MR6 (Rak *et al.* 2007b) was done using *KANMX4* DNA*-*cassettes [amplified with Low and Up primers (Table 2)] and genomic DNA was isolated from *aep1Δ*, *aep2Δ*, and *atp25Δ* deletion strains from Euroscarf. The deletions of *AEP1*, *AEP2*, and *ATP25* in MR6 were PCR-verified with Ver-KanMx primers (Table 2). Prior to deleting *AEP1*, *AEP2*, or *ATP25*, the MR6 strain was transformed with the pAM19 plasmid, which encodes the *PaAtp9-5* gene of *Podospora anserina* (here referred to as *ATP9-nuc*) (Bietenhader *et al.* 2012). In the presence of pAM19, *aep1Δ*, *aep2Δ*, and *atp25Δ* strains have a much less reduced propensity to produce ρ^−^/ρ^0^ cells lacking functional mtDNA. The ectopic integration of *ATP6* in mtDNA, with and without the *atp6*-L_173_P mutation, was done using a previously described procedure (Steele *et al.* 1996). As a first step, the *WT* and mutated *ATP6* genes flanked by 75 bp of the 5′-UTR and 118 bp of the 3′-UTR of *COX2* were PCR-amplified using GeneArt Invitrogen Gene Synthesis technology and cloned at the EcoRI site of pPT24 plasmid (Thorsness and Fox 1993), giving plasmids pRK49 and pRK77, respectively. These plasmids were introduced into mitochondria of the ρ^0^ strain DFS160 by biolistic transformation using the Particle Delivery Systems PDS-1000/He (BIO-RAD) as previously described (Bonnefoy and Fox 2001), giving the *petite* synthetic ρ^–^ strains RKY89-2 and RKY116-2. These strains were crossed with the strain RKY83 (Bader *et al.* 2020) in which the entire coding sequence of *ATP6* is replaced with *ARG8^m^* (*atp6::ARG8^m^*) and where the gene *COX2* is partially deleted (*cox2-62*). The RKY89-2 × RKY83 and RKY116-2 × RKY83 crosses produced clones (RKY112 and RKY117, respectively) that were arginine auxotrophic and able to grow in rich glycerol (YPGlyA). The ectopic integration of the *WT* or the mutated *ATP6* gene in strains RKY112 and RKY117 in the intergenic region of *COX2* was PCR-verified with primers oAtp6-2, oAtp6-4, o5′UTR2 and o5′UTR1 primers (Table 2). The modified mitochondrial genetic *loci* in strains RKY112 and RKY117 are schematically represented in Fig. 4a.

**Table 1.** Genotypes and sources of yeast strains.

Strain	Nuclear genotype	mtDNA	Source
MR6	*MAT* ***a*** *ade2-1 his3-11,15 trp1-1 leu2-3,112 ura3-1 arg8::HIS3 CAN1*	ρ^+^	Rak *et al.* (2007b)
D273-10B/60	*MATα met6*	ρ^o^	van Dijken *et al.* (2000)
DFS160	*MAT**α** leu2Δ ura3-52 ade2-101 arg8::URA3 kar1-1*	ρ^o^	Steele *et al.* (1996)
NB40-3C	*MAT**a** lys2 leu2-3,112 ura3-52 his3ΔHinDIII arg8::hisG*	ρ*^+^cox2-62*	Steele *et al.* (1996)
MR10 *(atp6Δ)*	*MAT**a** ade2-1 his3-11,15 trp1-1 leu2-3,112 ura3-1 CAN1 arg8::HIS3*	ρ*^+^ atp6::ARG8^m^*	Rak *et al.* (2007b)
SDC30	*MAT**α** leu2Δ ura3-52 ade2-101 arg8::URA3 kar1-1*	ρ*^−^ COX2 ATP6*	Rak *et al.* (2007b)
RKY20	*MAT**a** ade2-1 his3-11,15 trp1-1 leu2-3,112 ura3-1 arg8::HIS3 CAN1*	ρ*^+^ atp6-*L_173_P	Kucharczyk *et al.* (2009)
RKY12	*MATα leu2Δ ura3-52 ade2-101 arg8::URA3 kar1-1*	ρ*^−^ atp6-*L_173_P	Kucharczyk *et al.* (2009)
RKY26 *(atp9Δ)*	*MAT* ***a*** *ade2-1 his3-11,15 trp1-1 leu2-3,112 ura3-1 arg8::HIS3 CAN1*	ρ*^+^atp9::ARG8^m^*	Bietenhader *et al.* (2012)
AMY10 *(atp9Δ)*	*MAT* ***a*** *ade2-1 his3-11,15 trp1-1 leu2-3,112 ura3-1 arg8Δ::HIS3 CAN1/PaATP9-5 (ATP9-nuc)*	ρ^+^*atp9::ARG8^m^*	Bietenhader *et al.* (2012)
RKY83	*MAT* ***a*** *ade2-1 his3-11,15 trp1-1 leu2-3,112 ura3-1 arg8::HIS3 CAN1*	ρ*^+^cox2-62 atp6::ARG8^m^*	Bader *et al.* (2020)
RKY89	*MAT**α** leu2Δ ura3-52 ade2-101 arg8∷ URA3 kar1-1*	ρ*^−^5*′*-UTR_COX2_ ATP6^WT^*	Bader *et al.* (2020)
RKY112	*MAT* ***a*** *ade2-1 his3-11,15 trp1-1 leu2-3,112 ura3-1 arg8::HIS3 CAN1*	ρ*^+^5*′*-UTR_COX2_ ATP6^WT^*	Bader *et al.* (2020)
RKY116	*MAT**α** leu2Δ ura3-52 ade2-101 arg8:: URA3 kar1-1*	ρ*^−^ 5*′*-UTR_COX2_ atp6-L_173_P*	This study
RKY117	*MAT* ***a*** *ade2-1 his3-11,15 trp1-1 leu2-3,112 ura3-1 arg8::HIS3 CAN1*	ρ^+^ *5*′*-UTR_COX2_ atp6-L_173_P*	This study
RKY39	*MAT* ***a*** *ade2-1 his3-11,15 trp1-1 leu2-3,112 ura3-1 CAN1 arg8:: HIS3*	ρ^+^ *atp6-*W_126_R	Kucharczyk *et al.* (2013)
RKY39-R1G	*MAT* ***a*** *ade2-1 his3-11,15 trp1-1 leu2-3,112 ura3-1 CAN1 arg8:: HIS3*	ρ^+^ *atp6-*R_126_I	Kucharczyk *et al.* (2013)
RKY39-R5G	*MAT* ***a*** *ade2-1 his3-11,15 trp1-1 leu2-3,112 ura3-1 CAN1 arg8:: HIS3*	ρ^+^ *atp6-*R_126_G	Kucharczyk *et al.* (2019)
RKY39-6G	*MAT* ***a*** *ade2-1 his3-11,15 trp1-1 leu2-3,112 ura3-1 CAN1 arg8::HIS3*	ρ*^+^atp6-*W_126_R R_169_I	Kucharczyk *et al.* (2019)
RKY39-R16G	*MAT* ***a*** *ade2-1 his3-11,15 trp1-1 leu2-3,112 ura3-1 CAN1 arg8:: HIS3*	ρ^+^ *atp6-*R_126_K	Kucharczyk *et al.* (2019)
AKY36	*MAT* ***a*** *ade2-1 his3-11,15 trp1-1 leu2-3,112 ura3-1 arg8::HIS3 atp1::KANMX4 CAN1*	ρ^*+*^	This study
AKY37	*MAT* ***a*** *ade2-1 his3-11,15 trp1-1 leu2-3,112 ura3-1 arg8::HIS3 atp1::KANMX4 CAN1*	ρ*^+^atp6-*L_173_P	This study
AKY40	*MAT* ***a*** *ade2-1 his3-11,15 trp1-1 leu2-3,112 ura3-1 arg8::HIS3 atp11::KANMX4 CAN1*	ρ^*+*^	This study
AKY41	*MAT* ***a*** *ade2-1 his3-11,15 trp1-1 leu2-3,112 ura3-1 arg8::HIS3 atp11::KANMX4 CAN1*	ρ*^+^atp6-*L_173_P	This study
AKY42	*MAT* ***a*** *ade2-1 his3-11,15 trp1-1 leu2-3,112 ura3-1 arg8::HIS3 atp12::KANMX4 CAN1*	ρ^*+*^	This study
AKY43	*MAT* ***a*** *ade2-1 his3-11,15 trp1-1 leu2-3,112 ura3-1 arg8::HIS3 atp12::KANMX4 CAN1*	ρ*^+^atp6-*L_173_P	This study
AKY76	*MAT* ***a*** *ade2-1 his3-11,15 trp1-1 leu2-3,112 ura3-1 arg8::HIS3 fmc1::KANMX4 CAN1*	ρ^*+*^	This study
AKY77	*MAT* ***a*** *ade2-1 his3-11,15 trp1-1 leu2-3,112 ura3-1 arg8::HIS3 fmc1::KANMX4 CAN1*	ρ*^+^atp6-*L_173_P	This study
AKY60	*MAT* ***a*** *ade2-1 his3-11,15 trp1-1 leu2-3,112 ura3-1 arg8::HIS3 aep1::KANMX4 CAN1/PaATP9-5 (ATP9-nuc)*	ρ^*+*^	This study
AKY63	*MAT* ***a*** *ade2-1 his3-11,15 trp1-1 leu2-3,112 ura3-1 arg8::HIS3 aep1:: KANMX4 CAN1/PaAtp9-5 (ATP9-nuc)*	ρ*^+^atp6-*L_173_P	This study
AKY61	*MAT* ***a*** *ade2-1 his3-11,15 trp1-1 leu2-3,112 ura3-1 arg8::HIS3 aep2::KANMX4 CAN1/PaATP9-5 (ATP9-nuc)*	ρ^*+*^	This study
AKY64	*MAT* ***a*** *ade2-1 his3-11,15 trp1-1 leu2-3,112 ura3-1 CAN1 arg8::HIS3 aep2:: KANMX4/PaAtp9-5 (ATP9-nuc)*	ρ*^+^atp6-*L_173_P	This study
AKY65	*MAT* ***a*** *ade2-1 his3-11,15 trp1-1 leu2-3,112 ura3-1 arg8::HIS3/PaAtp9-5 (ATP9-nuc)*	ρ^*+*^	This study
AKY75	*MAT* ***a*** *ade2-1 his3-11,15 trp1-1 leu2-3,112 ura3-1 arg8::HIS3/PaAtp9-5 (ATP9-nuc)*	ρ*^+^atp6-*L_173_P	This study
AKY121	*MAT* ***a*** *ade2-1 his3-11,15 trp1-1 leu2-3,112 ura3-1 CAN1 arg8::HIS3 atp12::KANMX4/PaAtp9-5 (ATP9-nuc)*	ρ^*+*^	This study
FG146	*MAT* ***a*** *ade2-1 his3-11,15 trp1-1 leu2-3,112 ura3-1 CAN1 arg8::HIS3/PaAtp9-5 (ATP9-nuc)*	ρ*^+^atp6::ARG8^m^*	This study
FG151	*MAT* ***a*** *ade2-1 his3-11,15 trp1-1 leu2-3,112 ura3-1 CAN1 arg8::HIS3 atp12::KANMX4*	ρ*^+^atp6::ARG8^m^*	This study
FG152	*MAT* ***a*** *ade2-1 his3-11,15 trp1-1 leu2-3,112 ura3-1 arg8::HIS3 atp12::KANMX4/PaAtp9-5 (ATP9-nuc)*	ρ*^+^atp6::ARG8^m^*	This study
FG153	*MAT* ***a*** *ade2-1 his3-11,15 trp1-1 leu2-3,112 ura3-1 CAN1 arg8::HIS3 atp12::KANMX4*	ρ*^+^atp9::ARG8^m^*	This study
FG154	*MAT* ***a*** *ade2-1 his3-11,15 trp1-1 leu2-3,112 ura3-1 CAN1 arg8::HIS3 atp12::KANMX4/PaAtp9-5 (ATP9-nuc)*	ρ*^+^atp9::ARG8^m^*	This study

**Table 2.** Primers.

Name	Sequence
Atp11-Up	5′-GTGCCGTATCAGTAGTCGTAGAAGC-3′
Atp11-Low	5′GCTGCTGGGAGGTACTCAACATCAG-3′
Atp11-Ver	5′GTACTCATCGAGCACCCTTTGC-3′
Atp12-Up	5′-GTGTCCTGGCGTTTCTTAAGCTCAC-3′
Atp12-Low	5′-CACACGGAAGCTGTATCGCACTC-3′
Atp12-Ver	5′-GCTAGCTGCTGATTGACCATATCC-3′
Fmc1-Up	5′-GCTTGATACGTTTGGACAGTAGTTC-3′
Fmc1-Low	5′-TACTTCATTCTGGGATGCCTATC-3′
Fmc1-Ver	5′GCTAGTGCCAACTCGTCGTG-3′
Aep1-Up	5′-CGTAGCACTTTGTTGTTCCATGC-3′
Aep1-Low	5′-CATTTGTCGCAACGGAATTATCTG-3′
Aep1-Ver	5′-GGTTCACCCGATTTCACTGG-3′
Aep1-Kl	5′-GGGTCTTAGAAAATGATTACTACAG-3′
Aep2-Up	5′-CCTTTGTACCAAATATACTGAAG-3′
Aep2-Low	5′-CATCGTTTTAAAGTACAACTCC-3′
Aep2-Ver	5′ GCTTTACGATCCACATTCCC3′
Aep2-Kl	5′-GATTAATGTGGATAAATAGACTGG-3′
Atp25-Up	5′-CTAACCTCTCTTCTAATATACTTGC-3′
Atp25-Low	5′-GCATTCAGGTCTGAGTAATGAGC-3′
Atp25-Ver	5′-GAAATGGGGTCACAATCATCC-3′
*oAtp6-2*	5′-GTATGATTCCATACTCATTTGC-3′
*oAtp6-4*	5′-GCAAATGAGTATGGAATCATAC-3′
*o5′UTR2*	5′-CCATCTCCATCTGTAAATCCTAC-3′
*o5′UTR1*	5′-GAAGCGGGAATCCCGTAAGG-3′
*Verif-KanMx*	5′-GGATGTATGGGCTAAATGTACG-3′

### Construction of plasmids encoding *Aep1*, *Aep22*, *Atp25*, and *Atp25*-Cter

The *AEP1* and *AEP2* genes were amplified using primer pairs Aep1-KL/Aep1-Low and Aep2-KL/Aep2-Low, respectively (Table 2) and cloned into pGEM-T easy vector (Promega). The *AEP1* and *AEP2* sequences were cut off from the resulting plasmids with BamHI/PstI and XbaI/SalI, respectively, and cloned into Yep351 (Hill *et al.* 1986) under control of the *MET25* promoter, yielding plasmids pRK54 and pRK57, respectively (the plasmids used in this study are listed in Table 3). The plasmid pRK56 was constructed by cloning in Yep351 the SacI–HindIII fragment encoding Atp25 in the previously described plasmid pG29ST18 (Zeng *et al.* 2008). The plasmid pAK13 was constructed by cloning in pRS424 (Brachmann *et al.* 1998) the HindIII–BamHI fragment encoding Atp25-Cter in the previously described plasmid pG29ST23 (Zeng 2008). The pAK13 was digested by NotI and BamHI and ligated with mitochondrial targeting sequence present in the previously described plasmid pYX232 (Westermann and Neupert 2000), yielding plasmid pAK19.

**Table 3.** Plasmids.

Name	Characteristics	Source
pCM189	2µ, Tet-off, *URA3*	Gari *et al.* (1997)
pAM19	*PaATP9-5 (ATP9-nuc)* in pCM189	Bietenhader *et al.* (2012)
pPT24	*COX2* in pBluescript-KS	Thorsness and Fox (1993)
pRK49	*5*′*UTR_COX2_-ATP6^WT^-3*′*UTR_COX2_* in pPT24	Bader *et al.* (2020)
pRK77	*5*′*UTR_COX2_-ATP6^L173P^-3*′*UTR_COX2_* in pPT24	This study
Yep351	2µ, *LEU2*	Hill *et al.* (1986)
pRK54	2µ, *AEP1* in Yep351	This study
pRK57	2µ, *AEP2* in Yep351	This study
pG29ST32	2µ, *ATP25*-N, *LEU2*	Zeng *et al.* (2008)
pG29ST23	2µ, *ATP25*-C, *URA3*	Zeng *et al.* (2008)
pRK56	2µ, *ATP25* in Yep351	This study
pRS424	2µ, *TRP1*	Brachmann *et al.* (1998)
pAK13	*ATP25*-C in pRS424	This study
pAK19	*ATP25*-C (+mitochondrial targeting sequence) in pRS424	This study

### In vivo labeling of mitochondrial translation products

A previously described protocol (Barrientos *et al.* 2002) was used to assess neo-synthesis of the mtDNA-encoded proteins in yeast cells grown to early exponential phase (10^7^ cells/mL) in 10 mL of rich (YPGalA) or complete synthetic (CSM) galactose media. After centrifugation, the cells were washed twice with a low sulfate medium containing 2% galactose, supplemented with histidine, tryptophan, leucine, uracil, adenine, and arginine (50 mg/L each), and then incubated for 2 h at 28°C in 500 µL of the same medium (to starve the cells for endogenous cysteine and methionine). After inhibiting cytosolic protein synthesis for 5 min with 1 mM cycloheximide, 50 µCi of [^35^S]-methionine + [^35^S]-cysteine (Amersham Biosciences) was added, and the incubation was prolonged for 20 min. Total protein extracts were prepared and samples with the same level of incorporated radioactivity were separated by SDS-PAGE (Laemmli 1970) in 17.5% acrylamide gels (to separate Atp8 and Atp9) and in 12% acrylamide containing 4M urea and 25% glycerol (to separate Atp6, Cox3, Cox2, and cytochrome *b*). After electrophoretic migration, the gels were dried and the radioactive proteins were visualized by autoradiography using a PhosphorImager after 1-week of exposure.

### Northern blot

Northern blot analysis was done as described (Rak *et al.* 2007b). The RNAs, extracted from whole cells grown in YPGalA, were separated in a 1% (w/v) agarose-6% (v/v) formaldehyde gel in MOPS buffer (20 mM MOPS, 5 mM sodium acetate, 1 mM EDTA), and then transferred to a Nytran membrane (Schleicher & Schuell) and hybridized with DNA probes specific to *ATP9* (a PCR product amplified from MR6 DNA with primers 5′-AATAAGATATATAAATAAGTCC and 5′-GAATGTTATTAATTTAATCAAATGAG) and 21S RNA (a PCR product amplified with primers 21S.1: 5′-GTATAAGGTGTGAACTCTGCTCCAT and 21S.2: 5′-GGGGAGACAGTTGTTGTATCATTAC). The *ATP9* and *21S* RNA probes were labeled with [α-32P]-dCTP using the Prime-a-Gene Labelling System kit from Promega and purified on MicroSpin G-25 columns from Amersham Biosciences. Hybridizations were carried out in 50% (v/v) formamide, 5× SSPE, 0.5% SDS, 7% polyethylene glycol 5000, 5× Denhardt’s solution, 100 µg/mL carrier DNA at 42°C.

### BN- and SDS-PAGE

Mitochondria were isolated from yeast cells grown until middle exponential phase (2–3 × 10^7^ cells/mL) in rich galactose medium (YPGalA) at 28°C using the previously described enzymatic method (Guérin *et al.* 1979). Protein concentration was determined according to (Lowry *et al.* 1951) in the presence of 5% SDS. Blue native polyacrylamide gel electrophoresis (BN-PAGE) was done as described in (Schagger and von Jagow 1991). Briefly, a sample of mitochondria containing 200 µg of proteins was suspended in 100 µL of 30 mM HEPES, 150 mM potassium acetate, 12% glycerol, 2 mM 6-aminocaproic acid, 1 mM EGTA, 2% digitonin (from Sigma) buffered at pH 7.4 and supplemented with an inhibitor cocktail tablet (from Roche). After 30 min of incubation on ice, the extract was cleared by centrifugation (18,600 g, 4°C, 30 min), supplemented with 4.5 µL of loading dye (5% Serva Blue G-250, 750 mM 6-aminocaproic acid), and run in NativePAGE^TM^ 3-12% Bis-Tris Gel (Invitrogen). The proteins were transferred onto a polyvinylidene difluoride membrane and probed with 1:5,000 diluted polyclonal antibodies against Atp6/subunit *a*, Atp1/subunit α, and Atp9/subunit *c* (a gift from J. Velours). The tested proteins were revealed using peroxidase-labeled antibodies (Promega) at a 1:5,000 dilution and the ECL reagent of Pierce ECL Western Blotting Substrate (Thermo Scientific). SDS-PAGE analysis and immunological detection of proteins from isolated mitochondria or whole cells was performed as previously described (Schagger and von Jagow 1991). The open source program Image J (http://rsbweb.nih.gov/ij/) was used to quantify the immunological signals and statistical significance between samples was evaluated using Student’s *t*-test.

## Results

### Regulation of *ATP6*

#### Translation of assembly-defective subunit *6* variants is upregulated

**Fig. 1.**
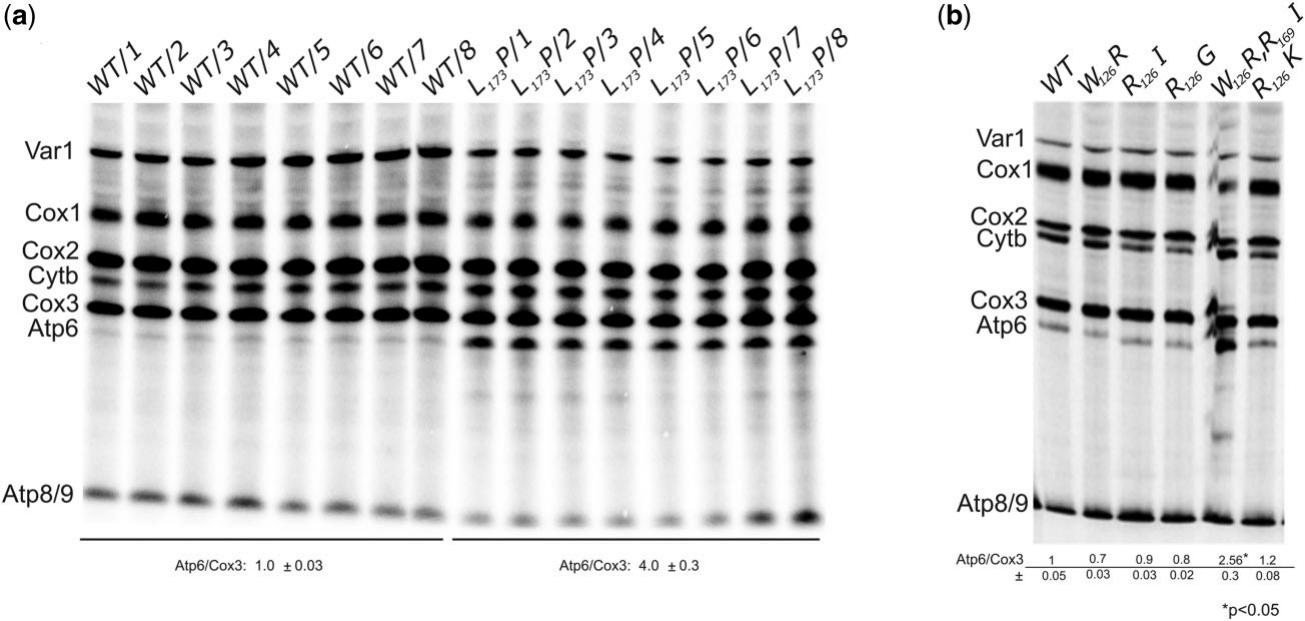
Mitochondrial translation in various subunit *6* mutants. a) The influence of the subunit *6* (Atp6) L_173_P variant on mitochondrial translation was investigated in 8 genetically independent recombinant *6*-L_173_P clones issued from crosses between RKY12 (a synthetic ρ^–^ with the *6*-L_173_P mutation) and MR10 [a ρ^+^ strain in which the coding sequence of *ATP6* is replaced with *ARG8^m^* (*atp6::ARG8^m^*)], in comparison to 8 genetically independent *WT* recombinant clones issued from crosses between SDC30 (a synthetic ρ^–^ containing the wild-type *ATP6* gene) and MR10 (see Table 1 for strain genotypes). b) Mitochondrial translation in 5 other subunit *6* mutants. Labeling of the mitochondrial translation products was performed in galactose grown cells during 20 min in the presence of [^35^S]-methionine and [^35^S]-cysteine and cycloheximide to block cytoplasmic translation. Total cellular protein extracts were then prepared and separated by SDS/PAGE in a 12% polyacrylamide gel containing 4 M urea and 25% glycerol. The gels were dried and the radiolabeled proteins were visualized using a PhosphorImager after 1-week exposure. The subunit *6* (Atp6) and Cox3 signals were quantified and compared to each other within each sample. The Atp6/Cox3 ratio was set to 1 for the *WT*.

In previous work, we observed that an assembly-defective mutant version of subunit *6* harboring a leucine-to-proline change at position 173 (L_173_P) in mature subunit *6* (which is produced after cleavage of the 10 N-terminal residues present in the precursor protein) was translated at a rate that was 2–3-fold faster than the wild-type protein [(Kucharczyk *et al.* 2009a); see below]. To ascertain that the L_173_P change was responsible for this effect, mitochondrial translation was herein investigated in 8 genetically independent recombinant *6*-L_173_P clones issued from crosses between RKY12 (a synthetic ρ^–^ with the *6*-L_173_P mutation, see Table 1) and MR10 [a ρ^+^ strain in which the coding sequence of *ATP6* is replaced with *ARG8^m^* (*atp6::ARG8^m^*), see Table 1], in comparison to 8 genetically independent *WT* recombinant clones issued from crosses between SDC30 (a synthetic ρ^–^ containing the wild-type *ATP6* gene, see Table 1) and MR10. Owing to the presence in RKY12 and SDC30 of the nuclear karyogamy delaying *kar1-1* mutation, it was possible to isolate the mitochondrial DNA recombinants in MR10 haploid background (which is the one of *WT* strain MR6). As a result, the 16 clones are isogenic except for the L_173_P mutation. Mitochondrial translation in these 16 clones was conducted with whole cells in the presence of cycloheximide (to block cytosolic translation) during a rather short time (20 min) after the addition of a mixture of radioactively labeled [^35^S]-methionine and [^35^S]-cysteine, a procedure widely used to evaluate the rate of synthesis of the mitochondrial translation products. As shown in Fig. 1a, subunit *6* was upregulated in the 8 L_173_P clones vs. the 8 reconstructed *WT* clones, which demonstrates that no other change in nuclear and mitochondrial DNA was involved in the increased rate of subunit *6* synthesis in the L_173_P clones. Two other subunit *6* variants, L_173_R (Rak *et al.* 2007a) and W_126_R (Kucharczyk *et al.* 2013), which assemble normally but are compromised functionally, were translated at the normal rate. In an effort to explain these findings, we investigated by pulse labeling the translation efficiency for 5 additional subunit *6* variants, harboring either single- or double-point mutations. We observed accelerated translation for 1 subunit *6* variant, while the 4 others were synthesized at the normal rate (Fig. 1b). Remarkably, in related work, we found that the strain showing enhanced subunit *6* synthesis was the only subunit *6* variant from this group of 5 that showed an assembly defect (Kucharczyk *et al.* 2019). Hence, it would appear that yeast accelerates the rate of subunit *6* translation when assembly of the ATP synthase stalls at the point where subunit *6* should be incorporated to complete the enzyme’s proton channel.

#### What is the mechanism behind accelerated synthesis of assembly-defective subunit *6*?

There is solid evidence in the literature that supports a model for ATP synthase biogenesis in which subunit *6* does not get incorporated until late in the process (Tzagoloff *et al.* 2004; Rak *et al.* 2007b). Hence, it was deemed logical to investigate some of the structural elements that would already be in place in the partially assembled enzyme for actions that promote enhanced expression of assembly-defective subunit *6* variants. This investigation was conducted with strains where the subunit *6 *L_173_P mutation was combined to genetic modifications leading to defects in the assembly of the F_1_ or the *9*_10_-ring, and we also tested the requirement for the 5′-UTR of *ATP6* by ectopically expressing the subunit *6* variant L_173_P from the 5′-UTR of *COX2*.

##### The F_1_

**Fig. 2.**
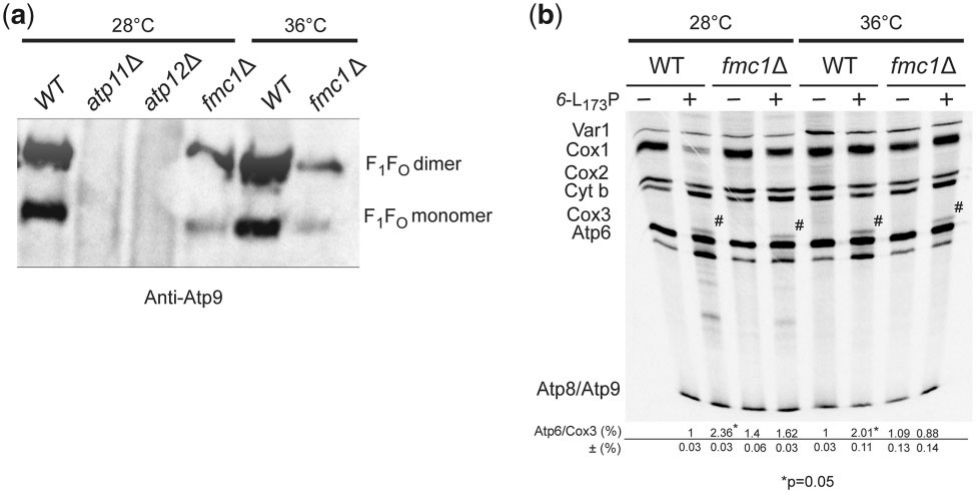
Upregulation of the subunit *6-L_173_*P variant is F_1_-dependent. a) ATP synthase in BN-gels. Mitochondria were isolated from the indicated strains grown in rich galactose at the indicated temperature. Proteins were extracted with digitonin (2 gr/gr protein) and separated by BN-PAGE on a 3–10% polyacrylamide gel (50 µg/lane). The proteins were transferred to a PVDF membrane and probed with antibodies against subunit 9. The F_1_–F_O_ monomers and dimers are identified on the right. b) Cells from *WT* and *fmc1Δ* yeasts with or without the subunit *6* mutation L_173_P were grown in rich galactose at 28°C or 36°C, as indicated, and then incubated for 20 min with [^35^S]-methionine and [^35^S]-cysteine in the presence of cycloheximide to block cytoplasmic translation. Total cellular protein extracts were then prepared and separated by SDS/PAGE in a 12% polyacrylamide gel containing 4 M urea and 25% glycerol (with a 30:0.8 ratio of acrylamide and *bis*-acrylamide). The gels were dried and the radiolabeled proteins were visualized using a PhosphorImager. The subunit *6* (Atp6) and Cox3 signals were quantified and compared to each other within each sample. The Atp6/Cox3 ratio was set to 1.0 for the *WT*. The identity of the band above Cox3 (designated by a hash sign) in the samples containing the subunit *6* variant L_173_P is unknown.

The importance of the F_1_ in the upregulation of the assembly-defective subunit *6* variant L_173_P was tested using the conditional strain *fmc1Δ*. This strain has a diminished capacity to assemble the F_1_ especially when grown at 36°C (5–10% vs*. WT*), whereas quite good levels of F_1_ accumulated in *fmc1Δ* cells grown at 28°C [(Lefebvre-Legendre *et al.* 2001), Fig. 2a]. At both temperatures, the strain *fmc1Δ* normally synthesizes the subunit *6* as well as the other proteins encoded by the mitochondrial DNA (Fig. 2b). The synthesis of the subunit *6* variant L_173_P was still stimulated in strain *fmc1Δ* grown at 28°C, although less efficiently than in a *WT* nuclear background, whereas the upregulation of subunit *6* was totally lost at 36°C. Hence, it appears that the mechanism responsible for the translation stimulation of the assembly-defective *6*-L_173_P variant is dependent on F_1_.

The gel of Fig. 2b shows a band of unknown identity (designated by a hash sign) above Cox3 in samples containing the subunit *6* variant L_173_P. As described below, it was detected in other experiments in which this mutant was used, and with a yeast strain lacking the *ATP6* gene (*atp6Δ*), indicating that this extra polypeptide was not some aberrant translation product derived from the modified *atp6 loci*.

##### Subunit 9

**Fig. 3.**
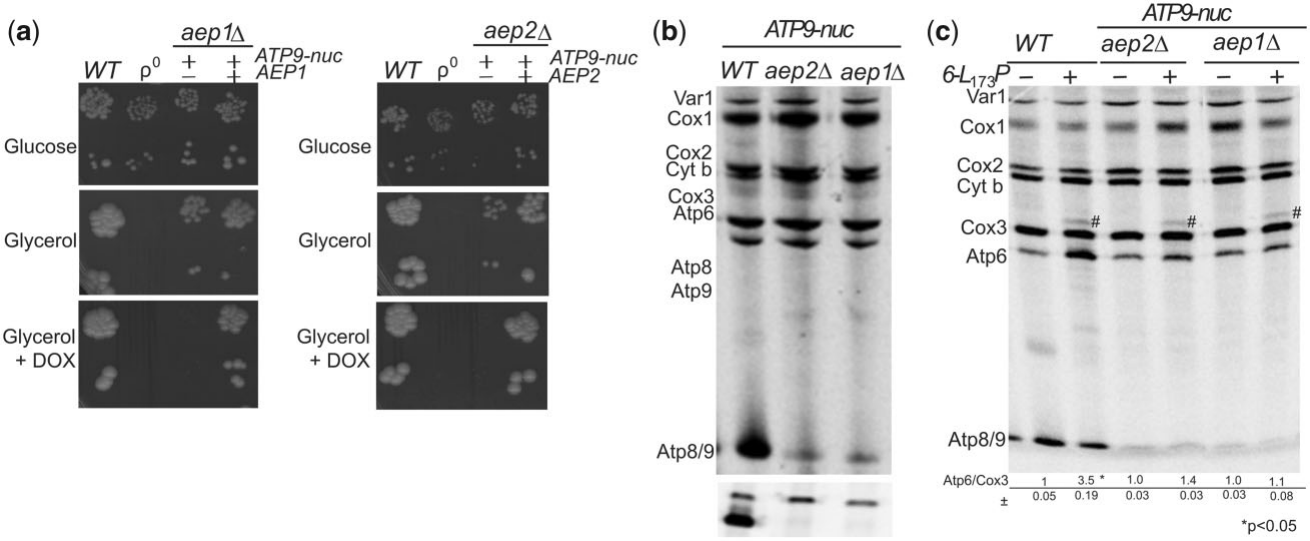
Upregulation of the subunit 6 variant L_173_P is subunit 9-dependent. a) Growth phenotypes. Fresh glucose cultures of the indicated strains were serially diluted and spotted on rich glucose and glycerol media with or without 5 µg/mL doxycycline (DOX) as indicated. The glucose and glycerol plates were scanned after 3 and 6 days of incubation at 28°C, respectively. b and c) In vivo labeling of mitochondrial translation products. Pulse labeling was performed for 20 min with [^35^S]-methionine + [^35^S]-cysteine in the presence of cycloheximide to block cytoplasmic translation, in cells freshly grown in rich galactose medium. Total protein extracts were then prepared and separated by SDS/PAGE in 2 different gels: (1) 12% polyacrylamide containing 4 M urea and 25% glycerol (to resolve Var1, Cox1, Cox2, Cytochrome *b*, Cox3, and Atp6); (2) 17.5% of polyacrylamide (to resolve Atp8 and Atp9). The 2 gels shown in b) were loaded with equal amounts of radioactivity from the same experiment. After drying of the gels under vacuum, the radiolabeled proteins were visualized using a PhosphorImager after one-week of exposure. The subunit *6* (Atp6) and Cox3 signals were quantified and compared to each other within each sample. The Atp6/Cox3 ratio was set to 1.0 for the *WT*. The identity of the band above Cox3 (designated by a hash sign) in the samples containing the *6*-L_173_P variant is unknown.

Yeast cells lacking the subunit *9* have a high propensity to lose the mitochondrial genome (Bietenhader *et al.* 2012), which makes it difficult to evaluate the importance of this protein in the upregulation of assembly-defective subunit *6*. To overcome this difficulty, we used *aep1Δ* and *aep2Δ* strains unable to synthesize subunit *9* and a previously characterized nuclear *ATP9* gene present in *P.* *anserina* [*PaAtp9-5* (Déquard-Chablat *et al.* 2011), here referred to as *ATP9-nuc*]. Although the protein product of *ATP9-nuc* (Atp9-nuc) shows a number of primary sequence differences with yeast subunit *9*, a yeast strain lacking the native mitochondrial *ATP9* gene (*atp9Δ*) had the ability to grow slowly on nonfermentable carbon sources upon transformation with a plasmid harboring *ATP9-nuc* (Bietenhader *et al.* 2012), showing that Atp9-nuc can assemble and function within the yeast ATP synthase albeit much less efficiently than subunit *9*. That Atp9-nuc only partially compensates for the absence of subunit *9* is not very surprising considering that *S. cerevisiae* is not optimized for ATP synthase biogenesis with a nucleus-encoded version of this protein that must be imported into mitochondria post-translationally, routed to the inner membrane, and assembled with the other yeast ATP synthase subunits. As expected, Atp9-nuc enabled *aep1Δ* and *aep2Δ* strains to grow slowly from respiratory carbon sources and when expression of Atp9-nuc was inhibited with doxycycline, they did not show any respiratory growth (Fig. 3a). The subunit *9* was not detected by pulse labeling in these 2 strains while the other proteins encoded by the mitochondrial DNA were all effectively synthesized (Fig. 3b). Importantly, although the F_O_ assembles poorly with Atp9-nuc, the F_1_ forms and accumulates efficiently (Bietenhader *et al.* 2012), which permitted us to evaluate the importance of the membrane motor of ATP synthase in the upregulation of the subunit *6* variant L_173_P without any interference due to a lack in F_1_. As shown in Fig. 3c, the L_173_P substitution did not stimulate subunit *6* synthesis in *aep1Δ* and *aep2Δ* mutants expressing Atp9-nuc indicating that subunit *9*, like the F_1_, is required for upregulating subunit *6* in response to mutations in this protein that compromise its assembly.

##### *The 5*′*-UTR of the ATP6 mRNA*

**Fig. 4.**
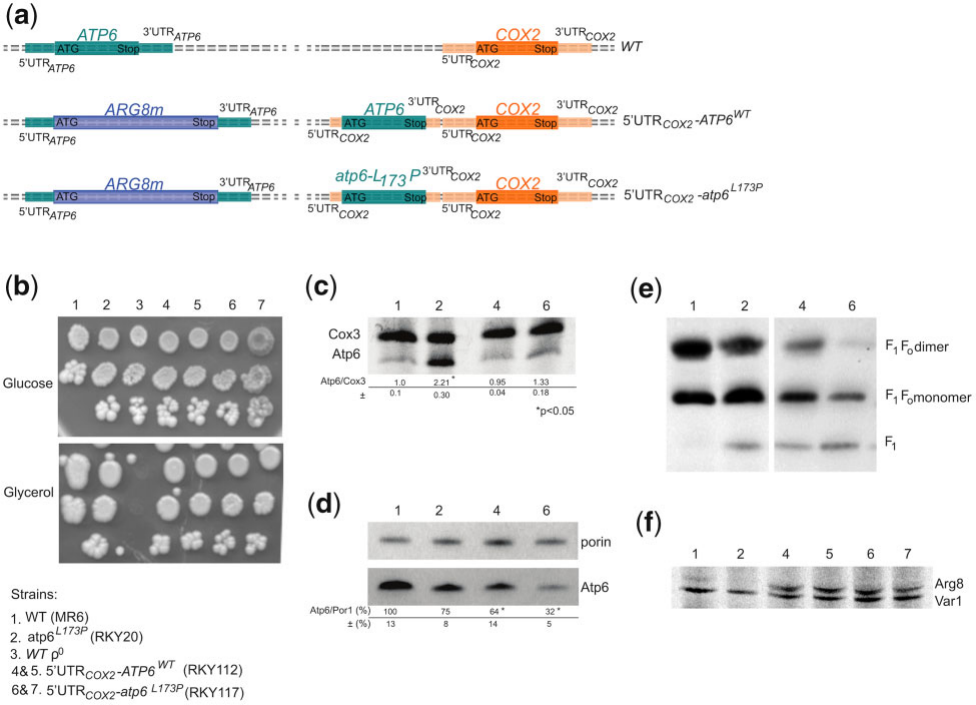
Pulse labeling of Atp6 and Cox3 and assembly of ATP synthase in strains expressing subunit 6 with and without the L_173_P mutation from the 5′UTR of COX2. a) Schema of mitochondrial genetic *loci*. As represented, the ectopic *ATP6* gene, with or without the L_173_P mutation, is located upstream of the *COX2* gene under control of the 5′-UTR and 3′-UTR of *COX2*, in a mitochondrial genome where the coding sequence of the native *ATP6* gene is replaced with *ARG8^m^*. The corresponding abbreviated genotypes (WT, 5′-UTR_COX2_-ATP6^WT^ and 5′-UTR_COX2_-ATP6^L173P^) are indicated on the right*.* b) Fresh glucose cultures of the strains with the indicated genotypes were serially diluted and spotted on rich glucose and rich glycerol media. The glucose and glycerol plates were scanned after 4 and 6 days of incubation at 28°C, respectively. c) In vivo labeling of Atp6 and Cox3. Pulse labeling of Atp6 and Cox3 was performed for 20 min with [^35^S]-methionine and [^35^S]-cysteine in the presence of cycloheximide to block cytoplasmic translation, in cells freshly grown in rich galactose medium. Total protein extracts were then prepared and separated by SDS/PAGE in a 12% polyacrylamide gel containing 4 M urea and 25% glycerol. After drying of the gel under vacuum, the radiolabeled proteins were visualized using a PhosphorImager after 1-week exposure. The subunit *6* (Atp6) and Cox3 signals were quantified and compared to each other within each sample. The Atp6/Cox3 ratio was set to 1.0 for the *WT*. d) Steady state levels of subunit *6*. Total cellular proteins samples (20 µg) extracted from the 4 analyzed strains grown in rich galactose medium were separated by SDS–PAGE in a 12% polyacrylamide gel. The proteins were transferred to a nitrocellulose membrane and probed with antibodies against subunit *6* (Atp6) and porin. The levels of subunit *6* relative to porin are expressed in % of *WT*. Standard deviation and statistical significance between the 2 strains are indicated (* corresponds to a *P*-value <0.05) e) Protein complexes were extracted from isolated mitochondria with digitonin (2 gr/gr protein) and separated by BN-PAGE in a 3–10% polyacrylamide gel. After their transfer to a PVDF membrane, they were probed with antibodies against α-F_1_. The F_1_F_O_ dimers and monomers, and free F_1_ are identified in the left-hand margin. f) In vivo labeling of Arg8 in strains RKY112 and RKY116. The rate of Arg8 synthesis in RKY112 and RKY116 from the 5′UTR of *ATP6* (*atp6::ARG8^m^*) relative to Var1 was probed by pulse labeling in whole galactose grown cells for 20 min with [^35^S]-methionine and [^35^S]-cysteine in the presence of cycloheximide to block cytoplasmic translation, with *WT* and RKY20 strains as controls that do not encode Arg8. Total protein extracts were then prepared and separated by SDS/PAGE in a 12% polyacrylamide gel containing 4 M urea and 25% glycerol. After drying of the gel under vacuum, the radiolabeled proteins were visualized using a PhosphorImager.

Expression of each yeast mitochondrial gene involves a peculiar 5′-UTR and specific translational activator protein(s) (Fox 2012; Herrmann *et al.* 2013; Ott *et al.* 2016). To test the implication of the 5′-UTR of *ATP6* in the upregulation of the assembly-defective subunit *6* variant L_173_P, we ectopically expressed *ATP6* with or without the L_173_P mutation from the 5′-UTR of *COX2* in a strain where the native *ATP6* gene is in-frame replaced with *ARG8m* (Rak *et al.* 2007b) (Fig. 4a). In the resulting strains (5′UTR_COX2_-*ATP6*^WT^ and 5′UTR_COX2_-*atp6*^L173P^), subunit *6* translation is activated by Pet111 (the translation activator of *COX2*) instead of Atp22 (the translation activator of *ATP6*). Both strains grew well on glycerol (Fig. 4b) and synthesized subunit *6* as effectively as in *WT* yeast (Fig. 4c), which indicated that the 5′-UTR of the *ATP6* mRNA is needed for stimulating translation of the subunit *6* variant L_173_P. Interestingly, despite a good rate of synthesis the ectopically expressed subunit *6* without the L_173_P mutation (5′UTR_COX2_-*ATP6*^WT^) was about 2 times less abundant at the steady-state than in *WT* cells expressing subunit *6* from its own 5′-UTR (Fig. 4d). While BN-PAGE analyses revealed only fully assembled ATP synthase dimers and monomers in mitochondria from the *WT*, those from the 5′UTR_COX2_-*ATP6*^WT^ strain additionally contain free F_1_ particles (Fig. 4e). The presence of free F_1_ is typically observed in strains where subunit *6* fails to assemble properly (Rak *et al.* 2007b), and it can therefore be inferred that the assembly of subunit *6* was less efficient when it was expressed from the 5′-UTR of *COX2* instead of its native 5′-UTR. Not surprisingly, a further decrease in the levels of subunit *6* was observed with the ectopic *atp6*-L_173_P gene (Fig. 4e), reflecting the deleterious consequences by its own of the leucine-to-proline change on the incorporation of subunit *6* into ATP synthase.

Both ectopic strains (5′UTR_COX2_-*ATP6*^WT^ and 5′UTR_COX2_-*atp6*^L173P^) express Arg8 from the 5′ UTR of ATP6 (Fig. 4a). As they are both deficient in the incorporation of subunit *6* into ATP synthase it was expected that Arg8 should be synthesized similarly in the 2 strains. This was indeed observed (Fig. 4f).

### Regulation of *ATP9*

#### Translation of subunit *9* is stimulated by *ATP9-nuc* and a lack in subunit *6*

**Fig. 5.**
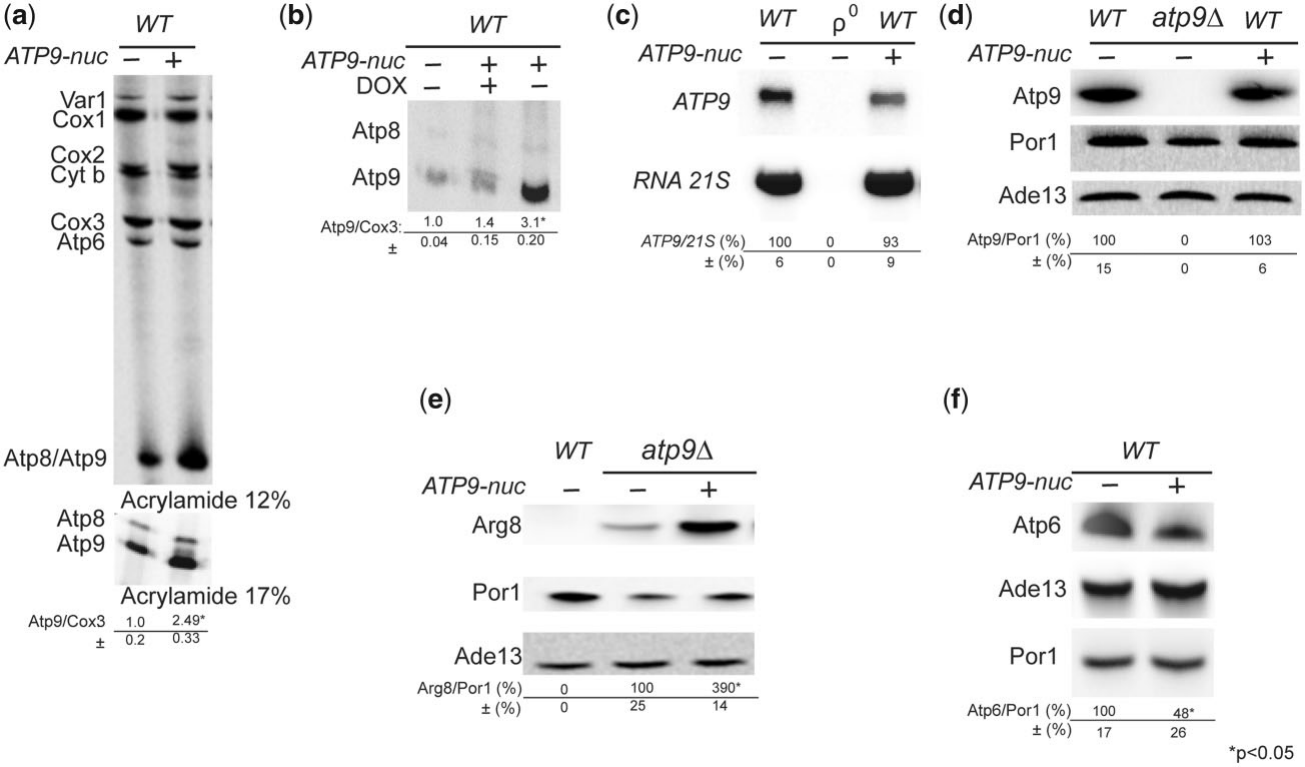
ATP9-nuc stimulates translation at the mitochondrial ATP9 locus. a) Influence of *ATP9-nuc* on the translation of mitochondrial gene products in *WT* yeast. Pulse labeling was performed in cells freshly grown in rich galactose medium for 20 min with [^35^S]-methionine and [^35^S]-cysteine in the presence of cycloheximide to block cytoplasmic translation. Total cellular proteins were then extracted and separated by SDS/PAGE in 2 different gels: (1) 12% polyacrylamide containing 4 M urea and 25% glycerol (to resolve Var1, Cox1, Cox2, Cytochrome *b*, Cox3, and Atp6); (2) 17.5% polyacrylamide (to resolve Atp8 and Atp9). The 2 gels were loaded with equal amounts of radioactivity from the same experiment. After drying of the gels under vacuum, the radiolabeled proteins were visualized using a PhosphorImager. The subunit *9* (Atp6) and Cox3 signals were quantified and compared to each other within each sample. The Atp9/Cox3 ratio was set to 1.0 for the *WT*. Standard deviation and statistical significance of the differences between strains is indicated (* corresponds to a *P*-value <0.05). b) Evaluation of subunit *9* synthesis in *WT* yeast after blocking expression of *ATP9-nuc* with 5 µg/mL doxycycline (DOX), using the same procedure as in a). c) Northern blot analyses. Total RNAs isolated from cells with the indicated genotypes (ρ^0^ is a derivative of the *WT* strain totally devoid of mtDNA) freshly grown in rich galactose medium were separated in a 1% agarose gel. After their transfer to a Nytran membrane, the RNAs were hybridized with ^32^P labeled DNA probes specific to *ATP9* and *21S RNA*. The *ATP9* mRNA signals were normalized to those corresponding to *21S RNA*. d–f) Steady state levels of proteins in cells. Total proteins extracted from cells with the indicated genotypes grown in rich galactose medium were separated by SDS–PAGE (20 µg/lane), transferred to a nitrocellulose membrane, and probed with antibodies against the indicated proteins. The levels of subunits *6* (Atp6), *9* (Atp9), and Arg8 (expressed from *atp9∷ARG8^m^*) are normalized to porin. Ade13 is used as a cytosolic protein marker. The symbol “*” indicates a statistically significant difference between samples (*P*-value <0.05).

Because of the primary sequence differences between the fungal Atp9-nuc and yeast subunit *9* proteins (Déquard-Chablat *et al.* 2011) and the poor capacity of Atp9-nuc to support the assembly of a functional ATP synthase in yeast cells lacking subunit *9* (Bietenhader *et al.* 2012), we reasoned that expressing Atp9-nuc in wild-type yeast could influence translation and/or assembly of the yeast subunit *9* protein. In support of this hypothesis, co-expressing Atp9-nuc and subunit *9* resulted in an accelerated rate of subunit *9* synthesis, whereas that of the other mitochondrial gene products was unaffected (Fig. 5a). This effect was lost when expression of the plasmid-borne fungal gene was inhibited with doxycycline (Fig. 5b), which demonstrated that Atp9-nuc was truly responsible for the increased rate of subunit *9* synthesis. The steady state levels of *ATP9* mRNA and its protein product were identical in untransformed *WT* and *WT* cells transformed with the Atp9-nuc gene (Fig. 5c and d). Cumulatively, these results showed that effect from Atp9-nuc was on translation, not transcription, and that the accelerated translation of subunit *9* did not lead to a stable accumulation of the overexpressed protein. One could hypothesize that the enhanced labeling of subunit *9* in *WT* expressing *ATP9-nuc* resulted from interactions with Atp9-nuc that stabilize subunit *9* and not from a higher rate of translation. At odds with this, the expression of Arg8 from the *atp9∷ARG8^m^* locus in *atp9Δ* yeast (in which the subunit *9* is thus absent) was also considerably stimulated by Atp9-nuc (Fig. 5e), which definitely demonstrates that the higher labeling of subunit *9* in *WT* yeast expressing Atp9-nuc was caused by a faster rate of synthesis of this protein.

**Fig. 6.**
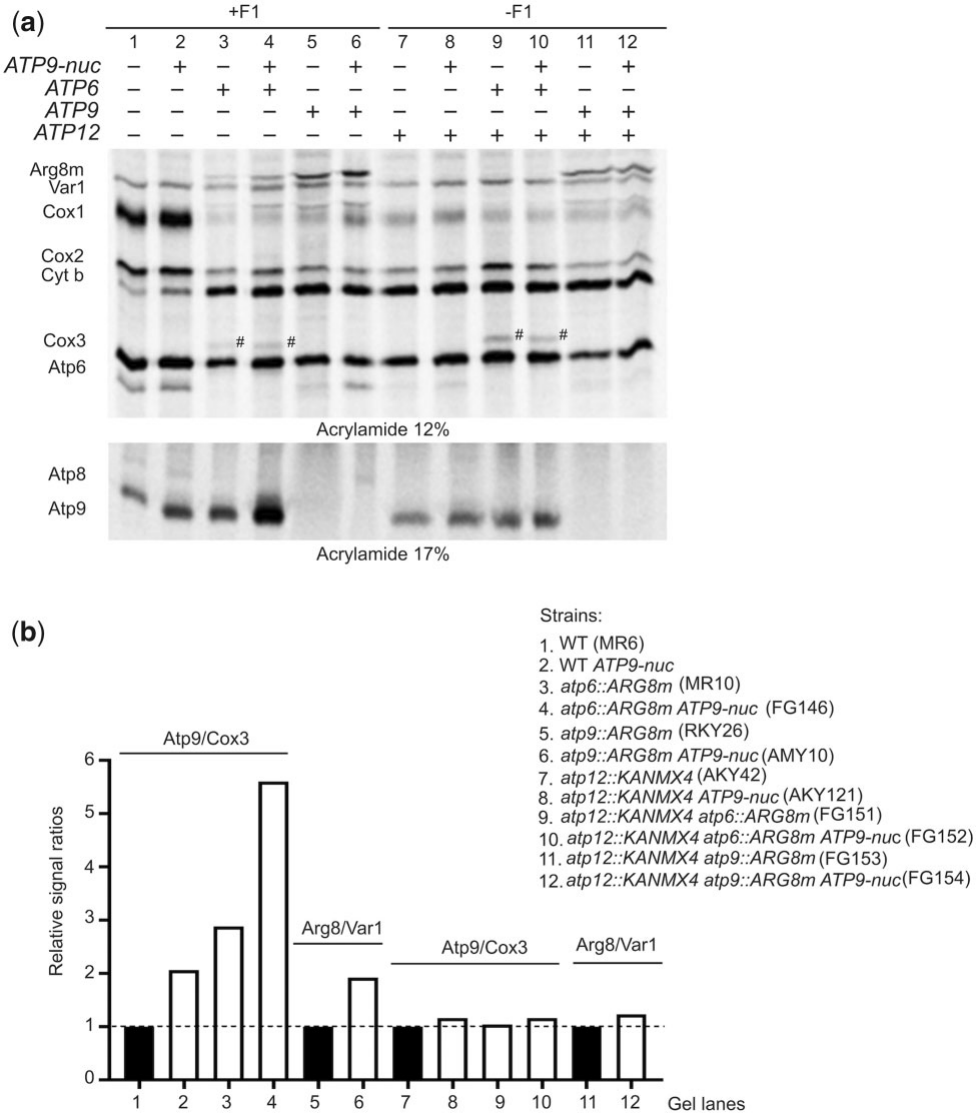
Upregulation of the mitochondrial *ATP9* locus by *ATP9-nuc* and/or a lack in *Atp6* is F_1_-dependent. a) Cells with the indicated genotypes were grown in rich galactose medium and their mitochondrially encoded proteins were radioactively labeled for 20 min with [^35^S]-methionine and [^35^S]-cysteine in the presence of cycloheximide to inhibit cytosolic translation. Total protein extracts were then prepared and resolved by SDS/PAGE in 2 types of gel loaded with the same amount of radioactivity (as in Fig. 2). After drying of the gels, the proteins were using a PhosphoImager. b) Quantification of subunit *9* and Arg8 expressed from the *ATP9* locus (*atp9∷ARG8^m^*). The levels of subunit *9* and Cox3 in each sample were quantified and compared to each other. Those in lanes 1–4 are compared to each other after setting to 1.0 the Atp9/Cox3 ratio lane 1. Those in lanes 7–10 are compared to each other after setting to 1.0 the Atp9/Cox3 ratio in lane 7. The levels of Arg8 and Var1 were quantified in lanes 5, 6, 11, 12. The Arg8/Var1 ratio in lane 5 was set to 1.0 and compared to the one in lane 6. The Arg8/Var1 ratio in lane 11 was set to 1.0 and compared to the one in lane 12.

While subunit *6* was synthesized normally in *WT* cells expressing the fungal gene for Atp9-nuc (Fig. 5a), the steady state levels of subunit *6* were diminished by about 50% relative to untransformed *WT* (Fig. 5f). In view of the fact that unassembled subunit *6* is highly susceptible to proteolytic degradation (Lefebvre-Legendre *et al.* 2001; Kucharczyk *et al.* 2009b; Rak *et al.* 2009; Godard *et al.* 2011; Kabala *et al.* 2014; Su *et al.* 2020), the lack of subunit *6* induced by Atp9-nuc suggested that Atp9-nuc results in ATP synthase formation defects (see below for a possible explanation)*.* Possibly, the lack in subunit *6* was responsible for, or contributed to the stimulation of subunit *9* translation. Indeed, subunit *9* translation was found stimulated in a strain lacking the *ATP6* gene (*atp6Δ*) [(Rak *et al.* 2007b), see also Fig. 6a, lane 3 and b). However, transforming *atp6Δ* yeast with Atp9-nuc resulted in a much higher (5.6-fold) stimulation of subunit *9* synthesis (Fig. 6a, lane 4), indicating that the partial lack in subunit *6* in *WT* cells expressing Atp9-nuc was not alone responsible for the enhanced rate of subunit *9* translation.

#### Upregulation of *ATP9* induced by *ATP9-nuc* and/or a lack in subunit *6* is F_1_-dependent

The rate of subunit *9* synthesis was found unaffected in strains lacking the α or β subunit of F_1_ or unable to assemble these proteins due to the absence of Atp11 or Atp12, indicating that the F_1_ does not influence subunit *9* synthesis (Rak *et al.* 2009, 2011). However, in the absence of Atp12, subunit *9* synthesis was no longer upregulated in strains expressing *ATP9-nuc* and/or lacking subunit *6* (Fig. 6a, lanes 2, 3, 4 vs. 8, 9, 10, respectively, and b), indicating that expression of subunit *9* is not F_1_-independent. The F_1_-dependency of the *ATP9* locus expression is further supported by the enhanced labeling of Arg8 from the *atp9∷ARG8^m^* allele (*atp9Δ*) in the presence of *ATP9-nuc* (Figs. 5e and 6a, lanes 5 vs. 6, and b) and the loss of this effect when the *ATP12* gene was deleted (Fig. 6a, lanes 11 vs. 12, and b). Another interesting observation is the much higher labeling of Arg8 in *atp9Δ* vs*. atp6Δ* yeasts (Fig. 6a, lanes 3 vs. 5), which is not very surprising due to the presence of 10 subunits *9* for 1 subunit *6* in ATP synthase.

The poor labeling of Cox1 in lanes 3–12 results from the lack of functional ATP synthase. This effect was observed in yeast ATP synthase defective mutants as long as there are no proton leaks through the F_O_ (Duvezin-Caubet *et al.* 2003; Rak *et al.* 2007a, 2007b; Kucharczyk *et al.* 2009a, 2009b, 2010), which is the case in all the mutant strains with a reduced Cox1 labeling in Fig. 6. Based on these observations, it has been argued that the biogenesis of complex IV is modulated by the activity of F_O_ (Su *et al.* 2019).

#### Subunit *9* is more susceptible to degradation and fails to assemble in the absence of F_1_

**Fig. 7.**
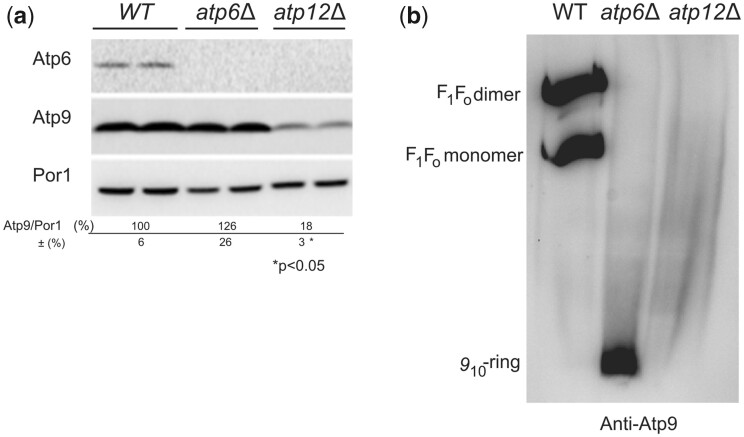
Influence of a lack in *Atp12* or subunit *6* on the steady state levels and assembly of subunit 9. a) Steady states levels of subunit *9* in *atp12Δ* and *atp6Δ* yeasts. Total protein extracts from *WT*, *atp6Δ*, and *atp12Δ* strains grown in rich galactose medium were prepared and separated by SDS–PAGE in a 12% polyacrylamide gel. The proteins were transferred to a nitrocellulose membrane, and probed with antibody against subunit *6* (Atp6), subunit *9* (Atp9), and porin (Por1). The reported values (expressed as %WT) were calculated from 3 independent experiments. Statistical significance of the differences between samples is indicated. b) Assembly of subunit *9* in *atp12Δ* and *atp6Δ* yeasts. Mitochondria were isolated from *WT*, *atp6Δ*, and *atp12Δ* strains grown in rich galactose medium. Digitonin extracts containing the same quantity of proteins (50 µg) were prepared and separated in 3–10% polyacrylamide BN gel, transferred to a PVDF membrane, and probed with antibodies against subunit *9*. The F_1_–F_O_ monomers and dimers, and subunit *9*-ring are identified in the left-hand margin.

Despite a good rate of synthesis (Fig. 6a, lanes 1 vs. 7), the steady levels of subunit *9* were diminished by about 80% in *atp12Δ* vs*. WT* (Fig. 7a) and the residual subunit *9* proteins were not detected as oligomeric rings in BN gels as they were in *atp6Δ* yeast (Fig. 7b). These observations suggest that formation/stability of the subunit *9*-ring is F_1_-dependent (see below).

## Discussion

There is solid evidence in the literature supporting a model in which ATP synthase formation starts with the assembly of F_1_ followed by association with the subunit *9*-ring (*9*_10_-ring) and peripheral stalk subunits, and finally incorporation of subunit *6*, in a process that involves a number of helper proteins with specific actions (Tzagoloff *et al.* 2004; Ackerman and Tzagoloff 2005; Rak *et al.* 2007b; Kucharczyk *et al.* 2008; Rak *et al.* 2009). In contrast, very few studies have so far been devoted to the mechanisms allowing the ATP synthase subunits to be produced in the good stoichiometry from 2 cell compartments (the cytosol and the mitochondrial matrix), an issue we have addressed in the present study.

**Fig. 8.**
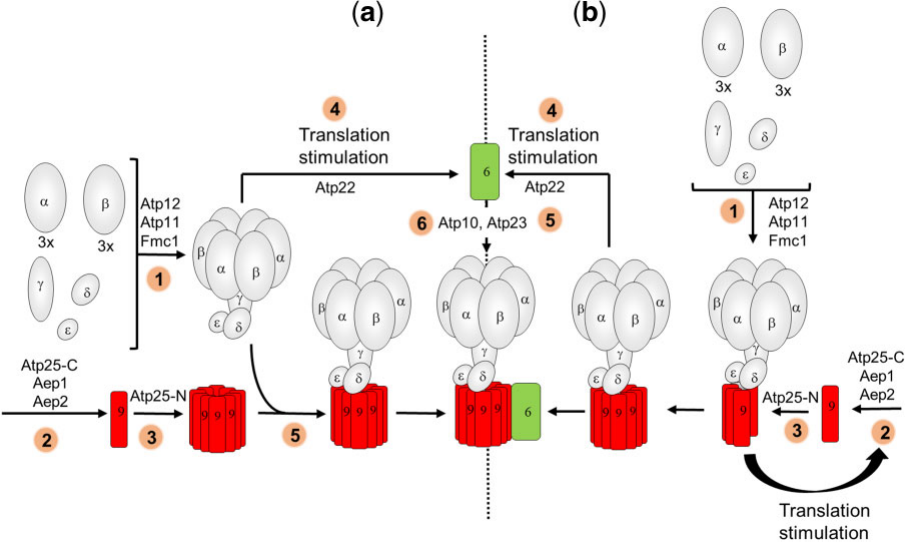
Model of assembly-dependent translation of subunits *6* and *9* of yeast ATP synthase. a) Previously reported model (Rak *et al.* 2009, 2011). 1: The subunits of F_1_ (α, β, γ, δ, ε) assemble with the help of Atp11, Atp12, and Fmc1. 2 and 3: The subunit *9* is produced and assembled with the help of Aep1, Aep2, Atp25-C, and Atp25-N independently of any other ATP synthase component. 4: The F_1_ alone is involved in the activation of subunit *6* translation by Atp22. 5: The F_1_ and the *9*_10_-ring associate to each other. 5: The subunit *6* with the help of Atp10 and Atp23 is incorporated into ATP synthase. b) Model based on the results reported in this study. 1: The subunits of F_1_ (α, β, γ, δ, ε) assemble with the help of Atp11, Atp12 and Fmc1. 2, 3: The synthesis and assembly of subunit *9* with the help of Aep1, Aep2, Atp25-C, and Atp25-N is F_1_-dependent. 4: The F_1_-*9*_10_ intermediate stimulates translation of subunit *6* by Atp22. 5: The subunit *6* with the help of Atp10 and Atp23 is incorporated into ATP synthase. The peripheral stalk subunits *8*, *4*, *i*, *j*, *f*, *d*, and OSCP of the ATP synthase monomer are not represented.

It has been reported that translation of subunits *6* and *8* is largely inhibited in strains with a virtual absence of F_1_ due to genetic ablation of α-F_1_ or β-F_1_ or 1 of 2 proteins (Atp11 and Atp12) that associate them to each other while the synthesis of subunit *9* is unaffected by the loss of F_1_ (Rak *et al.* 2009, 2011). On this basis, it was suggested that the F_1_ is the sole component of ATP synthase involved in the activation/stimulation of subunits *6* and *8* synthesis (by Atp22), and that the *9*_10_-ring forms separately, independently of any other ATP synthase component (Fig. 8a). This view is challenged by the present study.

We provide evidence that both the F_1_ and the *9*_10_-ring influence the synthesis of subunit *6*. Indeed, point mutations in this protein that partially compromise its assembly result in the acceleration of the rate of subunit *6* synthesis (Fig. 1), and this effect was lost in strains with a diminished capacity to assemble the F_1_ (Fig. 2) or the *9*_10_-ring (Fig. 3C), and in a strain expressing subunit *6* from the 5′UTR of the *COX2* gene (Fig. 4). These data suggest the existence of an assembly-dependent regulatory loop in which functional interaction of Atp22 [the translational activator of subunit *6* (Zeng *et al.* 2007a)] with the 5′-UTR of the *ATP6* mRNA involves both the F_1_ and the *9*_10_-ring rather than F_1_ alone as was previously proposed (Rak *et al.* 2009, 2011) (Fig. 8B).

Interestingly, while wild-type subunit *6* was efficiently synthesized from the 5′-UTR of *COX2* (Fig. 4c), there was at the steady state about 50% less subunit *6* than in wild-type yeast (Fig. 4d) and defects in ATP synthase were observed as evidenced by the presence of free F_1_ in mitochondrial samples resolved by BN-PAGE (Fig. 4e). Possibly, when not produced from its native 5′-UTR, nascent subunit *6* interacts less efficiently with Atp10 and Atp23 [2 proteins specifically required to incorporate subunit *6* into ATP synthase (Ackerman and Tzagoloff 1990a; Osman *et al.* 2007; Zeng *et al.* 2007c), and this reduces the yield in assembly. This conclusion is in line with previous studies that have revealed the importance of 5′-UTRs in post-translational biogenesis of other yeast mtDNA-encoded proteins (Sanchirico *et al.* 1998; Gruschke and Ott 2010; Gruschke *et al.* 2012).

Somewhat intriguingly, while translation of subunits *6* and *8* is largely inhibited in strains with a virtual absence of F_1_ (Rak *et al.* 2009, 2011), it is fully preserved with a 90% drop in F_1_ in yeast cells lacking a protein (Fmc1) that stabilizes Atp12 at 36°C [(Lefebvre-Legendre *et al.* 2001), Fig. 2]. The present study provides an explanation for these observations. Indeed, Atp22 was barely detectable in *atp11Δ* and *atp12Δ* mitochondria, whereas it accumulates well in those from *fmc1Δ* yeast grown at 36°C (Supplementary Fig. 1). The block in subunit *6* synthesis in strains totally lacking the F_1_ is thus probably due to the absence of Atp22 in mitochondria. Consistently, subunit *6* synthesis was fully recovered in these strains upon transformation with a high copy number plasmid harboring *ATP22* (Rak and Tzagoloff 2009). The lack of Atp22 in *atp11Δ* and *atp12Δ* mitochondria might result from the extremely detrimental consequences of a total or virtual lack in F_1_. Indeed, these mitochondria have a very poor content in complexes III and IV (Ebner and Schatz 1973), are very unstable genetically (Ebner and Schatz 1973), energize very poorly the inner membrane (Lefebvre-Legendre *et al.* 2003), and show severe defects in protein import (Yuan and Douglas 1992), whereas mitochondria from *fmc1Δ* cells grown at 36°C are much less affected (Lefebvre-Legendre *et al.* 2001) and do not show defects in protein import (Aiyar *et al.* 2014). Thus, major mitochondrial damage rather than disruption of a specific F_1_-dependent regulatory mechanism seems responsible for the lack of subunits *6* and *8* synthesis in yeast cells totally devoid of F_1_.

In revealing that subunit *6* synthesis is modulated by both the F_1_ and the *9*_10_-ring, our study describes a novel paradigm for the regulation of subunit *6* in which the F_1_–*9*_10_ subcomplex rather than the F_1_ alone modulates the synthesis of this protein. This model is in line with the concept of assembly-dependent regulation loop developed for cytochrome *b* and Cox1 according to which the synthesis of these proteins is stimulated by their direct protein partners (the F_1_ is not a direct partner of subunit *6*). As the subunit *6* is incorporated after formation of the F_1_-*9*_10_ intermediate, in all the models thus far proposed (Tzagoloff *et al.* 2004; Ackerman and Tzagoloff 2005; Rak *et al.* 2007b, 2009; Kucharczyk *et al.* 2008), it makes more sense that this intermediate rather than the F_1_ alone signals the need for producing subunit *6* as a means to couple the synthesis of this protein to its assembly.

Unlike previous studies (Rak *et al.* 2009, 2011), we conclude that the subunit *9* is also part of an assembly-dependent regulatory loop. A first clue was the higher pulse labeling of subunit *9* without any change in the levels of the *ATP9* mRNA in *WT* yeast transformed with a nuclear *ATP9* (*PaAtp9-5*) gene from *P. anserina* (Déquard-Chablat *et al.* 2011), here referred to as *ATP9-nuc*, and the loss of this effect after genetic ablation of F_1_ (Figs. 5 and 6). As *ATP9-nuc* also stimulated expression of Arg8 in a strain where the coding sequence of *ATP9* was replaced with *ARG8m* (Figs. 5e and 6a, lanes 3 and 5, and Fig. 6b), it is evident that *ATP9-nuc* stimulated the rate of translation at the *ATP9* locus whether the subunit *9* was or not present.

The ability of Atp9-nuc to partially restore the capacity of *atp9*Δ cells to grow in respiratory media shows that it can interact and function within yeast ATP synthase, albeit less efficiently than subunit *9* (Bietenhader *et al.* 2012). In the absence of Atp25-Nter, a polypeptide required for assembling the *9*_10_-ring (Zeng *et al.* 2008), the compensation of *atp9*Δ yeast by Atp9-nuc is less efficient (Supplementary Fig. 2), indicating that Atp9-nuc can functionally interact with Atp25-Nter. As a result of the interactions between Atp9-nuc and Atp25-Nter, there is less Atp25-Nter available for the assembly of the *9*_10_-ring. This may be responsible for the reduced content of subunit *6* in *WT* expressing Atp9-nuc (50% vs*. WT*) despite a normal rate of synthesis of this protein. Indeed, when it cannot interact properly with its protein partners, the subunit *6* becomes highly susceptible to proteolytic degradation (Ackerman and Tzagoloff 1990a; Zeng *et al.* 2007b, 2008; Kucharczyk *et al.* 2009b; Godard *et al.* 2011). There is thus no doubt that expressing Atp9-nuc in *WT* yeast results in defects in the assembly of ATP synthase, and this is what possibly causes the upregulation of subunit *9*. Indeed, it is a reasonable assumption that the *9*_10_-ring forms progressively with the addition one by one of subunit *9* monomers and newly synthesized subunit *9* is provided until completion of the ring. In the presence of Atp9-nuc, incomplete subunit *9* oligomers will be more abundant and in response, subunit *9* is synthesized more rapidly. The enhanced rate of subunit *9* translation observed in the absence of subunit *6* [3–4× in *atp6Δ* yeast; 5.6× in *atp6Δ* yeast expressing Atp9-nuc (Fig. 6)] may be explained similarly. Indeed, when not wedged between the F_1_ and subunit *6*, the *9*_10_-ring is certainly less stable and dissociates more frequently.

Another interesting finding here reported is the high susceptibly to degradation of unassembled subunit *9*, either when it is produced in excess (in *WT* + Atp9-nuc, Fig. 5) or when the F_1_ is absent (in *atp12Δ* yeast, Fig. 7a). The residual subunit *9* polypeptides in *atp12Δ* yeast (20% vs*. WT*) could not be detected as oligomers in BN-gels (Fig. 7b), and newly assembled *9*_10_-ring could not be detected after pulse labeling in mitochondria isolated from a strain lacking the F_1_ (Rak *et al.* 2011). These observations indicate that the subunit *9* cannot assemble in the absence of F_1_. It has been proposed that the entire process of yeast mitochondrial gene expression occurs within large platforms called MIOREX that interact with the mitochondrial ribosome and proteins that help the assembly of the OXPHOS complexes (including those involved in subunit *9* biogenesis) (Kehrein *et al.* 2015a, 2015b). It is tempting from the data here reported to speculate that newly formed F_1_ interacts with this platform to facilitate co-translational insertion of subunit *9* within ATP synthase (Fig. 8b).

The present study reveals that the synthesis of subunits *6* and *9* is coupled to the incorporation of these proteins into ATP synthase by mechanisms similar to those involved in the regulation of complexes III and IV biogenesis. In addition to increasing the yield in assembly of these 2 proteins, these regulations are likely important to prevent accumulation of free F_O_ and F_1_ particles that can dissipate the mitochondrial membrane electrical potential and the main source of chemical energy of the cell.

## Data availability

Strains and plasmids are available upon request. The authors affirm that all data necessary for confirming the conclusions of the article are present within the article, figures, and tables.

Supplemental material is available at *GENETICS* online.

## Funding

This work was supported by the National Institute of Health (5R01GM111873-02) to LMS and J-PdR, the Agence Nationale de la Recherche (ANR-09-BLAN-00500) to J-PdR, and (ANR-10-IDEX-0002) to HB and the National Science Centre of Poland (UMO-2011/01/B/NZ1/03492) to RK.

## Conflicts of interest

None declared.

## Supplementary Material

iyac007_Supplementary_Data

## References

[iyac007-B1] Ackerman SH. Atp11p and Atp12p are chaperones for F(1)-ATPase biogenesis in mitochondria. Biochim Biophys Acta. 2002;1555(1–3):101–105. 12206899 10.1016/s0005-2728(02)00262-1

[iyac007-B2] Ackerman SH , Gatti DL, Gellefors P, Douglas MG, Tzagoloff A. ATP13, a nuclear gene of *Saccharomyces cerevisiae* essential for the expression of subunit 9 of the mitochondrial ATPase. FEBS Lett. 1991;278(2):234–238. 1825065 10.1016/0014-5793(91)80124-l

[iyac007-B3] Ackerman SH , Tzagoloff A. ATP10, a yeast nuclear gene required for the assembly of the mitochondrial F1-F0 complex. J Biol Chem. 1990a;265(17):9952–9959. 2141026

[iyac007-B4] Ackerman SH , Tzagoloff A. Identification of two nuclear genes (ATP11, ATP12) required for assembly of the yeast F1-ATPase. Proc Natl Acad Sci U S A. 1990b;87(13):4986–4990. 2142305 10.1073/pnas.87.13.4986 PMC54246

[iyac007-B5] Ackerman SH , Tzagoloff A. Function, structure, and biogenesis of mitochondrial ATP synthase. Prog Nucleic Acid Res Mol Biol. 2005;80:95–133. 16164973 10.1016/S0079-6603(05)80003-0

[iyac007-B6] Aiyar RS , Bohnert M, Duvezin-Caubet S, Voisset C, Gagneur J, Fritsch ES, Couplan E, von der Malsburg K, Funaya C, Soubigou F, et al Mitochondrial protein sorting as a therapeutic target for ATP synthase disorders. Nat Commun. 2014;5:5585. 25519239 10.1038/ncomms6585 PMC4284804

[iyac007-B7] Altamura N , Capitanio N, Bonnefoy N, Papa S, Dujardin G. The *Saccharomyces cerevisiae* OXA1 gene is required for the correct assembly of cytochrome c oxidase and oligomycin-sensitive ATP synthase. FEBS Lett. 1996;382(1–2):111–115. 8612730 10.1016/0014-5793(96)00165-2

[iyac007-B8] Arechaga I , Butler PJ, Walker JE. Self-assembly of ATP synthase subunit c rings. FEBS Lett. 2002;515(1–3):189–193. 11943219 10.1016/s0014-5793(02)02447-x

[iyac007-B9] Asher EB , Groudinsky O, Dujardin G, Altamura N, Kermorgant M, Slonimski PP. Novel class of nuclear genes involved in both mRNA splicing and protein synthesis in *Saccharomyces cerevisiae* mitochondria. Mol Gen Genet. 1989;215(3):517–528. 2651895 10.1007/BF00427051

[iyac007-B10] Bader G , Enkler L, Araiso Y, Hemmerle M, Binko K, Baranowska E, De Craene J-O, Ruer-Laventie J, Pieters J, Tribouillard-Tanvier D, et al Assigning mitochondrial localization of dual localized proteins using a yeast Bi-Genomic Mitochondrial-Split-GFP. Elife. 2020;9:e56649. 10.7554/eLife.56649 PMC7358010 32657755

[iyac007-B11] Barrientos A , Korr D, Tzagoloff A. Shy1p is necessary for full expression of mitochondrial COX1 in the yeast model of Leigh’s syndrome. EMBO J. 2002;21(1–2):43–52. 11782424 10.1093/emboj/21.1.43 PMC125806

[iyac007-B12] Barrientos A , Zambrano A, Tzagoloff A. Mss51p and Cox14p jointly regulate mitochondrial Cox1p expression in *Saccharomyces cerevisiae*. EMBO J. 2004;23(17):3472–3482. 15306853 10.1038/sj.emboj.7600358 PMC516630

[iyac007-B13] Barros MH , Tzagoloff A. Aep3p-dependent translation of yeast mitochondrial ATP8. Mol Biol Cell. 2017;28(11):1426–1434. 28404747 10.1091/mbc.E16-11-0775 PMC5449143

[iyac007-B14] Bietenhader M , Martos A, Tetaud E, Aiyar RS, Sellem CH, Kucharczyk R, Clauder-Münster S, Giraud M-F, Godard F, Salin B, et al Experimental relocation of the mitochondrial ATP9 gene to the nucleus reveals forces underlying mitochondrial genome evolution. PLoS Genet. 2012;8(8):e1002876. 22916027 10.1371/journal.pgen.1002876 PMC3420929

[iyac007-B15] Bonnefoy N , Fox TD. Genetic transformation of *Saccharomyces cerevisiae* mitochondria. Methods Cell Biol. 2001;65:381–396. 11381605 10.1016/s0091-679x(01)65022-2

[iyac007-B16] Brachmann CB , Davies A, Cost GJ, Caputo E, Li J, Hieter P, Boeke JD. Designer deletion strains derived from *Saccharomyces cerevisiae* S288C: a useful set of strains and plasmids for PCR-mediated gene disruption and other applications. Yeast. 1998;14(2):115–132. 9483801 10.1002/(SICI)1097-0061(19980130)14:2<115::AID-YEA204>3.0.CO;2-2

[iyac007-B17] Chen W , Dieckmann CL. Cbp1p is required for message stability following 5'-processing of COB mRNA. J Biol Chem. 1994;269(24):16574–16578. 8206974

[iyac007-B18] Christianson T , Rabinowitz M. Identification of multiple transcriptional initiation sites on the yeast mitochondrial genome by in vitro capping with guanylyltransferase. J Biol Chem. 1983;258(22):14025–14033. 6315717

[iyac007-B19] Déquard-Chablat M , Sellem CH, Golik P, Bidard F, Martos A, Bietenhader M, di Rago J-P, Sainsard-Chanet A, Hermann-Le Denmat S, Contamine V, et al Two nuclear life cycle-regulated genes encode interchangeable subunits c of mitochondrial ATP synthase in *Podospora anserina*. Mol Biol Evol. 2011;28(7):2063–2075. 21273631 10.1093/molbev/msr025

[iyac007-B20] Dieckmann CL , Staples RR. Regulation of mitochondrial gene expression in *Saccharomyces cerevisiae*. Int Rev Cytol. 1994;152:145–181. 8206703 10.1016/s0074-7696(08)62556-5

[iyac007-B21] Duvezin-Caubet S , Caron M, Giraud MF, Velours J, di Rago JP. The two rotor components of yeast mitochondrial ATP synthase are mechanically coupled by subunit delta. Proc Natl Acad Sci U S A. 2003;100(23):13235–13240. 14581615 10.1073/pnas.2135169100 PMC263764

[iyac007-B22] Ebner E , Schatz G. Mitochondrial assembly in respiration-deficient mutants of *Saccharomyces cerevisiae*. 3. A nuclear mutant lacking mitochondrial adenosine triphosphatase. J Biol Chem. 1973;248(15):5379–5384. 4358614

[iyac007-B23] Ellis TP , Helfenbein KG, Tzagoloff A, Dieckmann CL. Aep3p stabilizes the mitochondrial bicistronic mRNA encoding subunits 6 and 8 of the H+-translocating ATP synthase of *Saccharomyces cerevisiae*. J Biol Chem. 2004;279(16):15728–15733. 14742425 10.1074/jbc.M314162200

[iyac007-B24] Finnegan PM , Ellis TP, Nagley P, Lukins HB. The mature AEP2 gene product of *Saccharomyces cerevisiae*, required for the expression of subunit 9 of ATP synthase, is a 58 kDa mitochondrial protein. FEBS Lett. 1995;368(3):505–508. 7635208 10.1016/0014-5793(95)00727-q

[iyac007-B25] Finnegan PM , Payne MJ, Keramidaris E, Lukins HB. Characterization of a yeast nuclear gene, AEP2, required for accumulation of mitochondrial mRNA encoding subunit 9 of the ATP synthase. Curr Genet. 1991;20(1–2):53–61. 1718609 10.1007/BF00312765

[iyac007-B26] Fontanesi F. Mechanisms of mitochondrial translational regulation. IUBMB Life. 2013;65(5):397–408. 23554047 10.1002/iub.1156

[iyac007-B27] Foury F , Roganti T, Lecrenier N, Purnelle B. The complete sequence of the mitochondrial genome of *Saccharomyces cerevisiae*. FEBS Lett. 1998;440(3):325–331. 9872396 10.1016/s0014-5793(98)01467-7

[iyac007-B28] Fox TD. Translational control of endogenous and recoded nuclear genes in yeast mitochondria: regulation and membrane targeting. Experientia. 1996;52(12):1130–1135. 8988256 10.1007/BF01952112

[iyac007-B29] Fox TD. Mitochondrial protein synthesis, import, and assembly. Genetics. 2012;192(4):1203–1234. 23212899 10.1534/genetics.112.141267 PMC3512135

[iyac007-B30] Gari E , Piedrafita L, Aldea M, Herrero E. A set of vectors with a tetracycline-regulatable promoter system for modulated gene expression in *Saccharomyces cerevisiae*. Yeast. 1997;13(9):837–848. 9234672 10.1002/(SICI)1097-0061(199707)13:9<837::AID-YEA145>3.0.CO;2-T

[iyac007-B31] Godard F , Tetaud E, Duvezin-Caubet S, di Rago JP. A genetic screen targeted on the FO component of mitochondrial ATP synthase in *Saccharomyces cerevisiae*. J Biol Chem. 2011;286(20):18181–18189. 21454598 10.1074/jbc.M110.214825 PMC3093890

[iyac007-B32] Gruschke S , Kehrein K, Römpler K, Gröne K, Israel L, Imhof A, Herrmann JM, Ott M. Cbp3-Cbp6 interacts with the yeast mitochondrial ribosomal tunnel exit and promotes cytochrome b synthesis and assembly. J Cell Biol. 2011;193(6):1101–1114. 21670217 10.1083/jcb.201103132 PMC3115798

[iyac007-B33] Gruschke S , Ott M. The polypeptide tunnel exit of the mitochondrial ribosome is tailored to meet the specific requirements of the organelle. Bioessays. 2010;32(12):1050–1057. 20967780 10.1002/bies.201000081

[iyac007-B34] Gruschke S , Römpler K, Hildenbeutel M, Kehrein K, Kühl I, Bonnefoy N, Ott M. The Cbp3-Cbp6 complex coordinates cytochrome b synthesis with bc(1) complex assembly in yeast mitochondria. J Cell Biol. 2012;199(1):137–150. 23007649 10.1083/jcb.201206040 PMC3461508

[iyac007-B35] Guérin B , Labbe P, Somlo M. Preparation of yeast mitochondria (*Saccharomyces cerevisiae*) with good P/O and respiratory control ratios. Methods Enzymol. 1979;55:149–159. 379498 10.1016/0076-6879(79)55021-6

[iyac007-B36] Hell K , Neupert W, Stuart RA. Oxa1p acts as a general membrane insertion machinery for proteins encoded by mitochondrial DNA. EMBO J. 2001;20(6):1281–1288. 11250894 10.1093/emboj/20.6.1281 PMC145526

[iyac007-B37] Herrmann JM , Neupert W, Stuart RA. Insertion into the mitochondrial inner membrane of a polytopic protein, the nuclear-encoded Oxa1p. EMBO J. 1997;16(9):2217–2226. 9171337 10.1093/emboj/16.9.2217 PMC1169824

[iyac007-B38] Herrmann JM , Woellhaf MW, Bonnefoy N. Control of protein synthesis in yeast mitochondria: the concept of translational activators. Biochim Biophys Acta. 2013;1833(2):286–294. 22450032 10.1016/j.bbamcr.2012.03.007

[iyac007-B39] Hildenbeutel M , Hegg EL, Stephan K, Gruschke S, Meunier B, Ott M. Assembly factors monitor sequential hemylation of cytochrome b to regulate mitochondrial translation. J Cell Biol. 2014;205(4):511–524. 24841564 10.1083/jcb.201401009 PMC4033779

[iyac007-B40] Hill JE , Myers AM, Koerner TJ, Tzagoloff A. Yeast/*E. coli* shuttle vectors with multiple unique restriction sites. Yeast. 1986;2(3):163–167. 3333305 10.1002/yea.320020304

[iyac007-B41] Jia L , Dienhart MK, Stuart RA. Oxa1 directly interacts with Atp9 and mediates its assembly into the mitochondrial F1Fo-ATP synthase complex. Mol Biol Cell. 2007;18(5):1897–1908. 17344477 10.1091/mbc.E06-10-0925 PMC1855041

[iyac007-B42] Kabala AM , Lasserre JP, Ackerman SH, di Rago JP, Kucharczyk R. Defining the impact on yeast ATP synthase of two pathogenic human mitochondrial DNA mutations, T9185C and T9191C. Biochimie. 2014;100:200–206. 24316278 10.1016/j.biochi.2013.11.024

[iyac007-B43] Kehrein K , Moller-Hergt BV, Ott M. The MIOREX complex–lean management of mitochondrial gene expression. Oncotarget. 2015a;6(19):16806–16807. 26220710 10.18632/oncotarget.4783 PMC4627264

[iyac007-B44] Kehrein K , Schilling R, Moller-Hergt BV, Wurm CA, Jakobs S, Lamkemeyer T, Langer T, Ott M. Organization of mitochondrial gene expression in two distinct ribosome-containing assemblies. Cell Rep. 2015b;10:843–853. 25683707 10.1016/j.celrep.2015.01.012

[iyac007-B45] Kucharczyk R , Dautant A, Gombeau K, Godard F, Tribouillard-Tanvier D, di Rago J-P. The pathogenic MT-ATP6 m.8851T>C mutation prevents proton movements within the n-side hydrophilic cleft of the membrane domain of ATP synthase. Biochim Biophys Acta Bioenerg. 2019;1860(7):562–572. 31181185 10.1016/j.bbabio.2019.06.002

[iyac007-B46] Kucharczyk R , Ezkurdia N, Couplan E, Procaccio V, Ackerman SH, Blondel M, di Rago J-P. Consequences of the pathogenic T9176C mutation of human mitochondrial DNA on yeast mitochondrial ATP synthase. Biochim Biophys Acta. 2010;1797(6–7):1105–1112. 20056103 10.1016/j.bbabio.2009.12.022 PMC2891117

[iyac007-B47] Kucharczyk R , Giraud M-F, Brèthes D, Wysocka-Kapcinska M, Ezkurdia N, Salin B, Velours J, Camougrand N, Haraux F, di Rago J-P, et al Defining the pathogenesis of human mtDNA mutations using a yeast model: the case of T8851C. Int J Biochem Cell Biol. 2013;45(1):130–140. 22789932 10.1016/j.biocel.2012.07.001

[iyac007-B48] Kucharczyk R , Rak M, di Rago JP. Biochemical consequences in yeast of the human mitochondrial DNA 8993T>C mutation in the ATPase6 gene found in NARP/MILS patients. Biochim Biophys Acta. 2009;1793(5):817–824. 19269308 10.1016/j.bbamcr.2009.02.011

[iyac007-B49] Kucharczyk R , Salin B, di Rago JP. Introducing the human Leigh syndrome mutation T9176G into *Saccharomyces cerevisiae* mitochondrial DNA leads to severe defects in the incorporation of Atp6p into the ATP synthase and in the mitochondrial morphology. Hum Mol Genet. 2009b;18(15):2889–2898. 19454486 10.1093/hmg/ddp226

[iyac007-B50] Kucharczyk R , Zick M, Bietenhader M, Rak M, Couplan E, Blondel M, Caubet S-D, di Rago J-P. Mitochondrial ATP synthase disorders: molecular mechanisms and the quest for curative therapeutic approaches. Biochim Biophys Acta. 2008;1793:186–199. 18620007 10.1016/j.bbamcr.2008.06.012

[iyac007-B51] Laemmli UK. Cleavage of structural proteins during the assembly of the head of bacteriophage T4. Nature. 1970;227(5259):680–685. 5432063 10.1038/227680a0

[iyac007-B52] Lefebvre-Legendre L , Balguerie A, Duvezin-Caubet S, Giraud M-F, Slonimski PP, Di Rago J-P. F1-catalysed ATP hydrolysis is required for mitochondrial biogenesis in *Saccharomyces cerevisiae* growing under conditions where it cannot respire. Mol Microbiol. 2003;47(5):1329–1339. 12603738 10.1046/j.1365-2958.2003.03371.x

[iyac007-B53] Lefebvre-Legendre L , Salin B, Schaëffer J, Brèthes D, Dautant A, Ackerman SH, di Rago J-P. Failure to assemble the alpha 3 beta 3 subcomplex of the ATP synthase leads to accumulation of the alpha and beta subunits within inclusion bodies and the loss of mitochondrial cristae in *Saccharomyces cerevisiae*. J Biol Chem. 2005;280(18):18386–18392. 15716275 10.1074/jbc.M410789200

[iyac007-B54] Lefebvre-Legendre L , Vaillier J, Benabdelhak H, Velours J, Slonimski PP, di Rago JP. Identification of a nuclear gene (FMC1) required for the assembly/stability of yeast mitochondrial F(1)-ATPase in heat stress conditions. J Biol Chem. 2001;276(9):6789–6796. 11096112 10.1074/jbc.M009557200

[iyac007-B55] Lowry OH , Rosebrough NJ, Farr AL, Randall RJ. Protein measurement with the Folin phenol reagent. J Biol Chem. 1951;193(1):265–275. 14907713

[iyac007-B56] Lytovchenko O , Naumenko N, Oeljeklaus S, Schmidt B, von der Malsburg K, Deckers M, Warscheid B, van der Laan M, Rehling P. The INA complex facilitates assembly of the peripheral stalk of the mitochondrial F1Fo-ATP synthase. EMBO J. 2014;33(15):1624–1638. 24942160 10.15252/embj.201488076 PMC4194097

[iyac007-B57] Mick DU , Vukotic M, Piechura H, Meyer HE, Warscheid B, Deckers M, Rehling P. Coa3 and Cox14 are essential for negative feedback regulation of COX1 translation in mitochondria. J Cell Biol. 2010;191(1):141–154. 20876281 10.1083/jcb.201007026 PMC2953447

[iyac007-B58] Osman C , Wilmes C, Tatsuta T, Langer T. Prohibitins interact genetically with Atp23, a novel processing peptidase and chaperone for the F1Fo-ATP synthase. Mol Biol Cell. 2007;18(2):627–635. 17135288 10.1091/mbc.E06-09-0839 PMC1783772

[iyac007-B59] Ott M , Amunts A, Brown A. Organization and regulation of mitochondrial protein synthesis. Annu Rev Biochem. 2016;85:77–101. 26789594 10.1146/annurev-biochem-060815-014334

[iyac007-B60] Ott M , Herrmann JM. Co-translational membrane insertion of mitochondrially encoded proteins. Biochim Biophys Acta. 2010;1803(6):767–775. 19962410 10.1016/j.bbamcr.2009.11.010

[iyac007-B61] Paul MF , Barrientos A, Tzagoloff A. A single amino acid change in subunit 6 of the yeast mitochondrial ATPase suppresses a null mutation in ATP10. J Biol Chem. 2000;275(38):29238–29243. 10867012 10.1074/jbc.M004546200

[iyac007-B62] Payne MJ , Finnegan PM, Smooker PM, Lukins HB. Characterization of a second nuclear gene, AEP1, required for expression of the mitochondrial OLI1 gene in *Saccharomyces cerevisiae*. Curr Genet. 1993;24(1–2):126–135. 8358819 10.1007/BF00324676

[iyac007-B63] Payne MJ , Schweizer E, Lukins HB. Properties of two nuclear pet mutants affecting expression of the mitochondrial oli1 gene of *Saccharomyces cerevisiae*. Curr Genet. 1991;19(5):343–351. 1833077 10.1007/BF00309594

[iyac007-B64] Pelissier P , Camougrand N, Velours G, Guerin M. NCA3, a nuclear gene involved in the mitochondrial expression of subunits 6 and 8 of the Fo-F1 ATP synthase of *S. cerevisiae*. Curr Genet. 1995;27(5):409–416. 7586026 10.1007/BF00311209

[iyac007-B65] Pelissier PP , Camougrand NM, Manon ST, Velours GM, Guerin MG. Regulation by nuclear genes of the mitochondrial synthesis of subunits 6 and 8 of the ATP synthase of *Saccharomyces cerevisiae*. J Biol Chem. 1992;267(4):2467–2473. 1531141

[iyac007-B66] Perez-Martinez X , Broadley SA, Fox TD. Mss51p promotes mitochondrial Cox1p synthesis and interacts with newly synthesized Cox1p. EMBO J. 2003;22(21):5951–5961. 14592991 10.1093/emboj/cdg566 PMC275423

[iyac007-B67] Pierrel F , Bestwick ML, Cobine PA, Khalimonchuk O, Cricco JA, Winge DR. Coa1 links the Mss51 post-translational function to Cox1 cofactor insertion in cytochrome c oxidase assembly. EMBO J. 2007;26(20):4335–4346. 17882260 10.1038/sj.emboj.7601861 PMC2034670

[iyac007-B68] Rak M , Gokova S, Tzagoloff A. Modular assembly of yeast mitochondrial ATP synthase. EMBO J. 2011;30(5):920–930. 21266956 10.1038/emboj.2010.364 PMC3049208

[iyac007-B69] Rak M , Su CH, Xu JT, Azpiroz R, Singh AM, Tzagoloff A. Regulation of mitochondrial translation of the ATP8/ATP6 mRNA by Smt1p. Mol Biol Cell. 2016;27(6):919–929. 26823015 10.1091/mbc.E15-09-0642 PMC4791136

[iyac007-B70] Rak M , Tetaud E, Duvezin-Caubet S, Ezkurdia N, Bietenhader M, Rytka J, di Rago J-P. A yeast model of the neurogenic ataxia retinitis pigmentosa (NARP) T8993G mutation in the mitochondrial ATP synthase-6 gene. J Biol Chem. 2007a;282(47):34039–34047. 17855363 10.1074/jbc.M703053200

[iyac007-B71] Rak M , Tetaud E, Godard F, Sagot I, Salin B, Duvezin-Caubet S, Slonimski PP, Rytka J, di Rago J-P. Yeast cells lacking the mitochondrial gene encoding the ATP synthase subunit 6 exhibit a selective loss of complex IV and unusual mitochondrial morphology. J Biol Chem. 2007b;282(15):10853–10864. 17261589 10.1074/jbc.M608692200

[iyac007-B72] Rak M , Tzagoloff A. F1-dependent translation of mitochondrially encoded Atp6p and Atp8p subunits of yeast ATP synthase. Proc Natl Acad Sci U S A. 2009;106(44):18509–18514. 19841266 10.1073/pnas.0910351106 PMC2774022

[iyac007-B75] Rodel G. Two yeast nuclear genes, CBS1 and CBS2, are required for translation of mitochondrial transcripts bearing the 5'-untranslated COB leader. Curr Genet. 1986;11:41–45. 3329045 10.1007/BF00389424

[iyac007-B76] Sanchirico ME , Fox TD, Mason TL. Accumulation of mitochondrially synthesized *Saccharomyces cerevisiae* Cox2p and Cox3p depends on targeting information in untranslated portions of their mRNAs. EMBO J. 1998;17(19):5796–5804. 9755179 10.1093/emboj/17.19.5796 PMC1170907

[iyac007-B77] Saraste M. Oxidative phosphorylation at the fin de siècle. Science. 1999;283(5407):1488–1493. 10066163 10.1126/science.283.5407.1488

[iyac007-B78] Schagger H , von Jagow G. Blue native electrophoresis for isolation of membrane protein complexes in enzymatically active form. Anal Biochem. 1991;199(2):223–231. 1812789 10.1016/0003-2697(91)90094-a

[iyac007-B79] Seshadri SR , Banarjee C, Barros MH, Fontanesi F. The translational activator Sov1 coordinates mitochondrial gene expression with mitoribosome biogenesis. Nucleic Acids Res. 2020;48(12):6759–6774. 32449921 10.1093/nar/gkaa424 PMC7337963

[iyac007-B80] Soto IC , Fontanesi F, Liu J, Barrientos A. Biogenesis and assembly of eukaryotic cytochrome c oxidase catalytic core. Biochim Biophys Acta. 2012;1817(6):883–897. 21958598 10.1016/j.bbabio.2011.09.005 PMC3262112

[iyac007-B81] Steele DF , Butler CA, Fox TD. Expression of a recoded nuclear gene inserted into yeast mitochondrial DNA is limited by mRNA-specific translational activation. Proc Natl Acad Sci U S A. 1996;93(11):5253–5257. 8643562 10.1073/pnas.93.11.5253 PMC39231

[iyac007-B82] Su X , Dautant A, Godard F, Bouhier M, Zoladek T, Kucharczyk R, di Rago J-P, Tribouillard-Tanvier D. Molecular basis of the pathogenic mechanism induced by the m.9191T>C mutation in mitochondrial ATP6 gene. Int J Mol Sci. 2020;21:5083. 32708436 10.3390/ijms21145083 PMC7404254

[iyac007-B83] Su X , Rak M, Tetaud E, Godard F, Sardin E, Bouhier M, Gombeau K, Caetano-Anollés D, Salin B, Chen H, et al Deregulating mitochondrial metabolite and ion transport has beneficial effects in yeast and human cellular models for NARP syndrome. Hum Mol Genet. 2019;28(22):3792–3804. 31276579 10.1093/hmg/ddz160

[iyac007-B84] Thorsness PE , Fox TD. Nuclear mutations in *Saccharomyces cerevisiae* that affect the escape of DNA from mitochondria to the nucleus. Genetics. 1993;134(1):21–28. 8514129 10.1093/genetics/134.1.21 PMC1205423

[iyac007-B85] Tzagoloff A , Barrientos A, Neupert W, Herrmann JM. Atp10p assists assembly of Atp6p into the F0 unit of the yeast mitochondrial ATPase. J Biol Chem. 2004;279(19):19775–19780. 14998992 10.1074/jbc.M401506200

[iyac007-B86] van Dijken JP , Bauer J, Brambilla L, Duboc P, Francois JM, Gancedo C, Giuseppin ML, Heijnen JJ, Hoare M, Lange HC, et al An interlaboratory comparison of physiological and genetic properties of four *Saccharomyces cerevisiae* strains. Enzyme Microb Technol. 2000;26:706–714. 10862876 10.1016/s0141-0229(00)00162-9

[iyac007-B87] Wang ZG , Ackerman SH. The assembly factor Atp11p binds to the beta-subunit of the mitochondrial F(1)-ATPase. J Biol Chem. 2000;275(8):5767–5772. 10681564 10.1074/jbc.275.8.5767

[iyac007-B88] Wang ZG , Sheluho D, Gatti DL, Ackerman SH. The alpha-subunit of the mitochondrial F(1) ATPase interacts directly with the assembly factor Atp12p. EMBO J. 2000;19(7):1486–1493. 10747017 10.1093/emboj/19.7.1486 PMC310218

[iyac007-B89] Wang ZG , White PS, Ackerman SH. Atp11p and Atp12p are assembly factors for the F(1)-ATPase in human mitochondria. J Biol Chem. 2001;276(33):30773–30778. 11410595 10.1074/jbc.M104133200

[iyac007-B90] Westermann B , Neupert W. Mitochondria-targeted green fluorescent proteins: convenient tools for the study of organelle biogenesis in *Saccharomyces cerevisiae*. Yeast. 2000;16(15):1421–1427. 11054823 10.1002/1097-0061(200011)16:15<1421::AID-YEA624>3.0.CO;2-U

[iyac007-B91] Woellhaf MW , Sommer F, Schroda M, Herrmann JM. Proteomic profiling of the mitochondrial ribosome identifies Atp25 as a composite mitochondrial precursor protein. Mol Biol Cell. 2016;27(20):3031–3039. 27582385 10.1091/mbc.E16-07-0513 PMC5063612

[iyac007-B92] Yuan H , Douglas MG. The mitochondrial F1ATPase alpha-subunit is necessary for efficient import of mitochondrial precursors. J Biol Chem. 1992;267(21):14697–14702. 1386080

[iyac007-B93] Zassenhaus HP , Martin NC, Butow RA. Origins of transcripts of the yeast mitochondrial var 1 gene. J Biol Chem. 1984;259(9):6019–6027. 6325458

[iyac007-B94] Zeng X , Barros MH, Shulman T, Tzagoloff A. ATP25, a new nuclear gene of *Saccharomyces cerevisiae* required for expression and assembly of the Atp9p subunit of mitochondrial ATPase. Mol Biol Cell. 2008;19(4):1366–1377. 18216280 10.1091/mbc.E07-08-0746 PMC2291438

[iyac007-B95] Zeng X , Hourset A, Tzagoloff A. The *Saccharomyces cerevisiae* ATP22 gene codes for the mitochondrial ATPase subunit 6-specific translation factor. Genetics. 2007a;175(1):55–63. 17110482 10.1534/genetics.106.065821 PMC1775023

[iyac007-B96] Zeng X , Kucharczyk R, di Rago JP, Tzagoloff A. The leader peptide of yeast Atp6p is required for efficient interaction with the Atp9p ring of the mitochondrial ATPase. J Biol Chem. 2007b;282(50):36167–36176. 17940284 10.1074/jbc.M705436200

[iyac007-B97] Zeng X , Neupert W, Tzagoloff A. The metalloprotease encoded by ATP23 has a dual function in processing and assembly of subunit 6 of mitochondrial ATPase. Mol Biol Cell. 2007c;18(2):617–626. 17135290 10.1091/mbc.E06-09-0801 PMC1783785

[iyac007-B98] Ziaja K , Michaelis G, Lisowsky T. Nuclear control of the messenger RNA expression for mitochondrial ATPase subunit 9 in a new yeast mutant. J Mol Biol. 1993;229(4):909–916. 8445655 10.1006/jmbi.1993.1095

