# Supplementary material for: Assembly-dependent translation of subunits 6 (Atp6) and 9 (Atp9) of ATP synthase in yeast mitochondria
Source: Genetics. 2022 Jan 20;220(3):iyac007. doi: 10.1093/genetics/iyac007 (PMC8893259; doi:10.1093/genetics/iyac007)
Supplement: iyac007_Supplementary_Data [file iyac007_supplementary_data.docx]

**Supplemental Figure 1**. *The steady levels of Atp22 in mitochondria of various F_1_ assembly-deficient mutants*. Mitochondria were isolated from *WT*, *atp11Δ*, *atp12Δ* and *fmc1Δ* strains grown in rich galactose medium at the indicated temperature. Samples containing 50 μg of proteins were separated by SDS–PAGE in a 12% polyacrylamide gel, transferred to a nitrocellulose membrane, and probed with antibodies against Atp22 and porin. The *hash sign* identifies a contaminant protein reacting with the Atp22 antibodies. The reported mean values in % of WT were calculated from 3 independent experiments.

**Supplemental Figure 2**. *Complementation of atp9Δ yeast by ATP9-nuc is improved by Atp25-Nter.* (A) Fresh cultures in glucose rich medium of the indicated strains were serially diluted and spotted on rich glucose and glycerol media with or without doxycycline (DOX). *ATP9-nuc* is the nuclear *PaAtp9-5* gene present in *P. anserina* (Déquard-Chablat *et al.* 2011) under control of the doxycycline-repressible Tet-Off promoter. The glucose and glycerol plates were scanned after 3 and 6 days of incubation at 28°C respectively (B) *In vivo* labeling of mitochondrial gene products. Cells from the indicated strain freshly grown in rich galactose medium were incubated for 20 min with [^35^S]-methionine and [^35^S]-cysteine in the presence of cycloheximide to block cytosolic translation. Total cellular extracts were then prepared and separated by SDS/PAGE in two different polyacrylamide gels with a 30:0.8 ratio of acrylamide and bis-acrylamide. Upper gel: 12% polyacrylamide, 4 M urea and 25% glycerol. Lower gel, 17.5% polyacrylamide. After drying of the gel under vacuum, the radiolabeled proteins were visualized using a PhosphorImager.
